# Smartphone-Delivered Ecological Momentary Interventions Based on Ecological Momentary Assessments to Promote Health Behaviors: Systematic Review and Adapted Checklist for Reporting Ecological Momentary Assessment and Intervention Studies

**DOI:** 10.2196/22890

**Published:** 2021-11-19

**Authors:** Kim Phuong Dao, Katrien De Cocker, Huong Ly Tong, A Baki Kocaballi, Clara Chow, Liliana Laranjo

**Affiliations:** 1 Westmead Applied Research Centre Faculty of Medicine and Health The University of Sydney Sydney Australia; 2 Capital Health Network Canberra Australia; 3 Institute for Resilient Regions Centre for Health Research University of Southern Queensland Springfield Central Australia; 4 School of Computer Science Faculty of Engineering & Information Technology University of Technology Sydney Sydney Australia

**Keywords:** ecological momentary assessment, ecological momentary intervention, behavior change, health behavior, mHealth, mobile health, smartphone apps, mobile phone

## Abstract

**Background:**

Healthy behaviors are crucial for maintaining a person’s health and well-being. The effects of health behavior interventions are mediated by individual and contextual factors that vary over time. Recently emerging smartphone-based ecological momentary interventions (EMIs) can use real-time user reports (ecological momentary assessments [EMAs]) to trigger appropriate support when needed in daily life.

**Objective:**

This systematic review aims to assess the characteristics of smartphone-delivered EMIs using self-reported EMAs in relation to their effects on health behaviors, user engagement, and user perspectives.

**Methods:**

We searched MEDLINE, Embase, PsycINFO, and CINAHL in June 2019 and updated the search in March 2020. We included experimental studies that incorporated EMIs based on EMAs delivered through smartphone apps to promote health behaviors in any health domain. Studies were independently screened. The PRISMA (Preferred Reporting Items for Systematic Reviews and Meta-Analyses) guidelines were followed. We performed a narrative synthesis of intervention effects, user perspectives and engagement, and intervention design and characteristics. Quality appraisal was conducted for all included studies.

**Results:**

We included 19 papers describing 17 unique studies and comprising 652 participants. Most studies were quasi-experimental (13/17, 76%), had small sample sizes, and great heterogeneity in intervention designs and measurements. EMIs were most popular in the mental health domain (8/17, 47%), followed by substance abuse (3/17, 18%), diet, weight loss, physical activity (4/17, 24%), and smoking (2/17, 12%). Of the 17 studies, the 4 (24%) included randomized controlled trials reported nonstatistically significant effects on health behaviors, and 4 (24%) quasi-experimental studies reported statistically significant pre-post improvements in self-reported primary outcomes, namely depressive (*P*<.001) and psychotic symptoms (*P*=.03), drinking frequency (*P*<.001), and eating patterns (*P*=.01). EMA was commonly used to capture subjective experiences as well as behaviors, whereas sensors were rarely used. Generally, users perceived EMIs to be helpful. Common suggestions for improvement included enhancing personalization, multimedia and interactive capabilities (eg, voice recording), and lowering the EMA reporting burden. EMI and EMA components were rarely reported and were not described in a standardized manner across studies, hampering progress in this field. A reporting checklist was developed to facilitate the interpretation and comparison of findings and enhance the transparency and replicability of future studies using EMAs and EMIs.

**Conclusions:**

The use of smartphone-delivered EMIs using self-reported EMAs to promote behavior change is an emerging area of research, with few studies evaluating efficacy. Such interventions could present an opportunity to enhance health but need further assessment in larger participant cohorts and well-designed evaluations following reporting checklists. Future research should explore combining self-reported EMAs of subjective experiences with objective data passively collected via sensors to promote personalization while minimizing user burden, as well as explore different EMA data collection methods (eg, chatbots).

**Trial Registration:**

PROSPERO CRD42019138739; https://www.crd.york.ac.uk/prospero/display_record.php?RecordID=138739

## Introduction

### Background

Mobile technologies have become popular approaches to promote behavior change and improve health outcomes, offering the ability to reach large populations in an easy, rapid, and low-cost manner [1,2]. Until recently, mobile behavior change interventions were limited to providing automated and predefined generic or minimally tailored messages, mainly based on estimates of *baseline* or *usual* behaviors and their determinants [3]. As people’s behaviors are driven by individual and contextual factors that vary across time [4,5], there is a need to make behavior change interventions that are more adaptive to the users’ evolving needs and context. Such an adaptive and dynamic intervention approach might help maintain participant engagement, sustain and support continued behavior change for longer durations, and thereby achieve greater health benefits [4-6].

Ecological momentary interventions (EMIs) are behavior change interventions that deliver support in real time, when most needed [7], for example, when the user is most likely to engage in unhealthy behaviors. To provide the information or treatment in real time and in real settings, EMIs are often based on repeated user reports collected via questionnaires, that is, ecological momentary assessments (EMAs) [8]. These EMA self-reports are usually real time or near real time and can focus on behaviors, contexts, emotional states, beliefs, attitudes, perceptions, exposures, events, or experiences in naturalistic settings (eg, “How are you feeling right now?”, “What are you doing right now?”, and “Are you near anyone smoking?”) [9]. EMAs originated in psychology a few decades ago, when these self-reports were primarily paper-based [8,9].

It has been suggested that tailoring EMIs based on EMAs may lead to higher user engagement and intervention effectiveness [7,10,11]. Given the ubiquity of smartphones [12,13], researchers are starting to explore the use of these mobile technologies to collect EMAs and deliver EMIs [14-17]. Previous systematic reviews of EMAs have focused on sedentary behavior, physical activity, and diet, mixing different EMA media for data collection, such as smartphones, PDAs (precursors of smartphones, now discontinued), and paper-and-pencil diaries [18-22]. The few existing systematic reviews on EMIs have focused on mental health and have also included studies with mixed media for EMIs, such as telephone, SMS text messaging, in-person counseling, computers, PDAs, and smartphones (a minority of included studies) [23-25]. To date, no studies have synthesized the current evidence on the use of smartphone-delivered EMIs using EMAs and their impact on health behaviors, user perspectives, or engagement.

### Objective

The overall objective of this study is to systematically review the evidence and characteristics of smartphone-delivered EMIs to promote behavior change, using self-reported EMAs, specifically (1) their effects on health behaviors in any health domain, (2) user engagement, and (3) user perspectives. Although not the original aim of this systematic review, another objective arose upon data extraction and analysis—developing a reporting checklist (adapted from an existing checklist [22]) to facilitate interpretation and comparison of findings and enhance transparency and replicability of future studies using EMAs and EMIs.

## Methods

The PRISMA (Preferred Reporting Items for Systematic Reviews and Meta-Analyses) guidelines were used when conducting and reporting this systematic review. The protocol was registered in PROSPERO (International Prospective Register of Systematic Reviews; CRD42019138739).

### Search Strategy for Identification of Studies

A literature search was conducted in June 2019 (and updated in March 2020) using MEDLINE (via PubMed interface), Embase, PsycINFO, and CINAHL. Search strings included a combination of free terms and controlled vocabulary when supported (complete search strategy available in Multimedia Appendix 1). The reference lists of relevant articles were also screened to ensure that all eligible studies were included. The authors were contacted if there was a need for any additional information about the included studies.

### Study Selection Criteria

The eligibility criteria were developed using the PICO (Participants, Intervention, Comparator, Outcomes; Multimedia Appendix 2). Participants included healthy individuals or patients with chronic conditions. We included all experimental studies that incorporated EMIs to improve health behaviors in any health domain. For the purposes of this review, an EMI must have been delivered in real time through smartphone apps and must have been based on data collected from users’ repeated reports in their natural context (ie, EMAs) and also via smartphone apps. Outcomes included any measures that illustrated the effects on health behavior changes (eg, changes in step counts and diet changes). Secondary outcomes included perspectives on EMIs and user engagement behaviors with different types of EMIs, including retention rate. No limiting criteria were used regarding comparison groups. Peer-reviewed studies published in English were included, and no restrictions were set regarding publication dates.

We excluded protocols, reviews, opinion pieces, and design and development papers without user evaluation of EMIs. Studies that used EMAs only for the purpose of data collection or outcome measurement were also excluded. Other exclusion criteria included interventions that relied solely on the automated data collected (eg, only through sensors and no user-reported EMAs) and interventions that were not based on data submitted by the participants (ie, EMAs) via smartphone apps or wearable devices.

### Screening, Data Extraction, and Synthesis

A pilot screening of the studies was completed before the actual screening process began. The title and abstract screening and full-text screening were conducted by 2 independent investigators. A third researcher resolved disagreements. Cohen κ was applied to measure the intercoder agreement in each screening phase.

An investigator extracted the information from the included studies into a standardized form, and another researcher reviewed the form for consistency. The data collected from each study included the first author, year of publication, location, health domain, intervention aim, study design and duration, participants’ settings and characteristics, EMA data collection characteristics (eg, type of information collected from participants, prompting design and frequency—following the CREMAS [Checklist for Reporting EMA Studies] reporting checklist [22]), intervention components (eg, app, website, and therapy sessions), smartphone-based EMI characteristics (eg, frequency), health-related outcomes, user’s perspectives regarding EMIs and EMAs, and user engagement. Behavior change techniques (BCTs) were coded by 2 researchers using the BCT taxonomy [26]. Included randomized controlled trials (RCTs) were appraised by 2 researchers using the Cochrane risk of bias tool [27]. Nonrandomized studies were appraised by 2 researchers using the *Risk Of Bias In Non-randomized Studies of Interventions* tool [28]. A narrative synthesis was conducted for all included studies.

## Results

### Description of Included Studies

**Figure 1:**
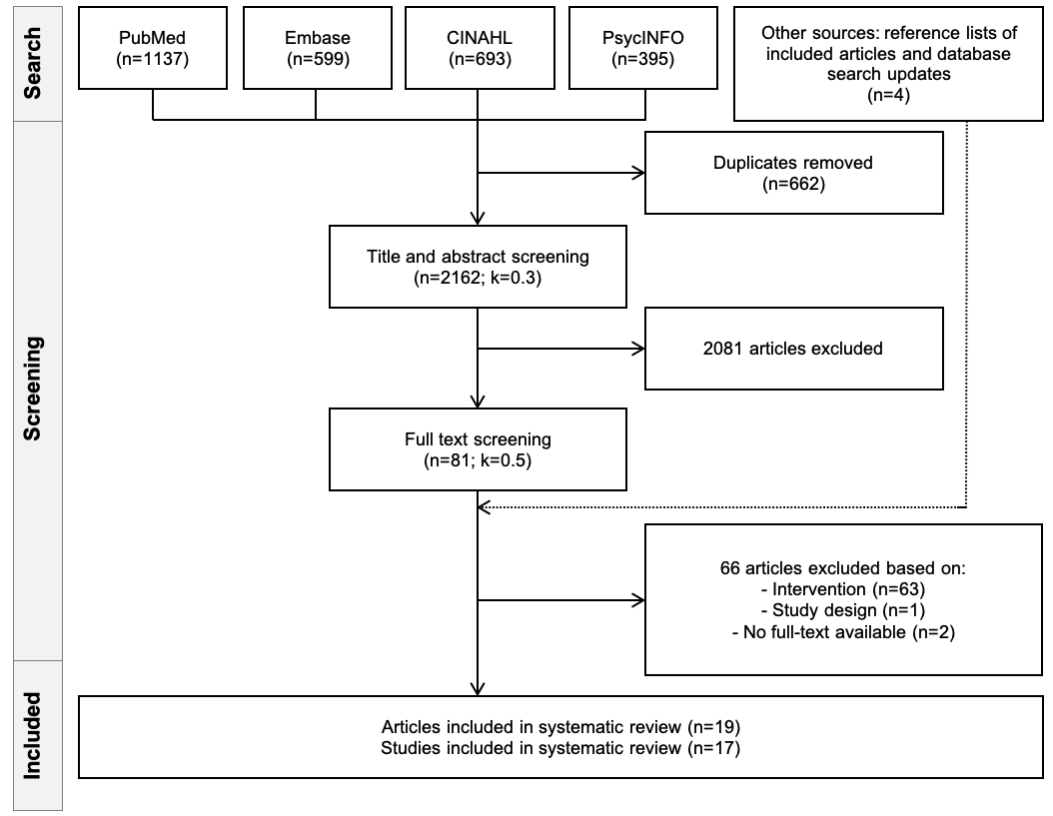
Flowchart of included studies.

The search returned 2824 results (Figure 1). Of the 2824 studies, after removing duplicates, 2162 (76.56%) studies underwent title and abstract screening. Of the 2162 studies, there were 81 (3.75%) studies for full-text screening; of the 81 studies, 66 (81%) were excluded for not meeting the inclusion criteria (reasons for exclusion are presented in Multimedia Appendix 3). Cohen κ scores were 0.3 and 0.5 for abstract and full-text screening, respectively. We included 15 papers from the original search and 4 additional papers from other sources (reference lists of included studies and database search updates), corresponding to 19 articles, describing 17 unique studies (Table 1).

The 17 included studies (19 papers) involved a total of 652 participants [29-47] (Table 1). Most studies (13/17, 76%; 15/19, 79% papers) were conducted in the United States [29-32,34,37-45,47]. Publication years ranged from 2011 to 2020 (13/17, 76% studies were published from 2016 onward). Study duration ranged from 2-15 weeks, and the average duration was 4 weeks. Sample size varied from 7-121 participants (mean 35.2, SD 33.3; 67% women). The health domains covered were: mental health [29-36], smoking cessation [37-39], and substance abuse control [40-42], as well as diet, weight loss, and physical activity [43,44,46,47]. Studies in the mental health domain mostly recruited patients from outpatient clinics diagnosed with a mental health problem (major depressive disorder [29], schizophrenia [34,36], bipolar disorder [31], and other conditions [30,32,35]), and only 1 study focusing on mood and anxiety management recruited participants without a diagnosis [33]. Studies focusing on smoking recruited participants from smoking cessation clinics [37-39]. Studies on substance abuse control recruited individuals currently in treatment for an alcohol disorder from the community [40], college students with problematic drinking [41], and marijuana users from primary care clinics [42]. Finally, studies on diet, weight loss, and physical activity recruited obese individuals undergoing assessment for bariatric surgery [43], overweight or obese participants from the community [44,45], university students interested in well-being [46], and African American women after breast cancer treatment, recruited from the community [47].

Of the 17 studies, there were 4 (24%; 5/19, 26% papers) RCTs [39,42,44,45,47] and 12 (71%; 13/19, 68% papers) quasi-experimental studies (all with a single-arm design [29-35,37,38,40,41,43,46] except for one with 2 arms [36]). Of the 17 studies, participant retention was reported in 14 (82%; 16/19, 84% papers) studies, ranging from 62.1%-100% in the intervention arm [29-31,34,36-47], with 11 (65%; 13/19, 68% papers) studies having retention rates >75% [29-31,34,36-38,40,41,44-47]. The risk of bias of the 4 RCTs was assessed as unclear for most of the *risk of bias* tool categories (Multimedia Appendix 4). Overall risk of bias in nonrandomized studies was assessed as *serious* for most studies (Multimedia Appendix 5).

**Table 1:** Characteristics of included studies.

Author, year, location			Intervention aim		Study design		Duration (weeks); Sample size (I^a^;C^b^); Age (years), mean (SD); Women (%);Characteristics		Study retention rate (%)		Intervention components		Health-related outcomes		*P* value
**Mental health**															
	Burns, 2011, United States [29]	Mood disorders management		Quasi-experimental, 1 arm		7; 8 (N/A^c^); 37.4 (12.2); 87%; Adults with major depressive disorder recruited on the web		87		App, website, phone coaching, emails, and sensors		Depressive symptoms decreased postintervention		<.001	
	Bush, 2014, United States [30]	Mood and anxiety disorders management		Quasi-experimental, 1 arm		2; 8 (N/A); NR^d^; 37%; Military personnel under treatment for behavioral health issues		100		App		NR		—^e^	
	Wenze, 2016, United States [31]	Bipolar disorder management		Quasi-experimental, 1 arm		12; 8 (N/A); 44 (11.6); 65%; Patients with bipolar disorder from a psychiatric hospital (inpatient and outpatient)		100		App and therapy sessions (4 weekly during 1 month)		NS^f^ Change in symptoms or adherence		—	
	Shrier, 2017, United States [32]	Impulse control disorder management		Quasi-experimental, 1 arm		4; 16 (N/A); 19.6 (NR); 100%; Primary care patients with depressive symptoms and at increased HIV risk		NR		App and therapy sessions		NR		—	
	Bakker, 2018, Australia [33]	Mood and anxiety disorders management		Quasi-experimental, 1 arm		4; 44 (N/A); 36 (13); 82%; Participants recruited on the web (no diagnosis needed)		NR		App		NR		—	
	Kreyenbuhl, 2019, United States [34]	Promote antipsychotic medication adherence		Quasi-experimental, 1 arm		2; 7 (N/A); 47.6 (10.4); 0%; African American men with schizophrenia from an outpatient mental health clinic		100		App and clinician appointment		Participants reported taking their antipsychotic medication in 100% of the adherence EMAs^g^ to which they responded		—	
	Vaessen, 2019, The Netherlands [35]	Psychotic disorders management		Quasi-experimental, 1 arm		Results for intervention arm of randomized controlled trial; 16 (N/A); NR; NR; First episode psychosis in the past 3 years, recruited from mental health clinics		NR		App and acceptance and commitment therapy sessions (weekly)		NR		—	
	Hanssen, 2020, The Netherlands [36]	Schizophrenia spectrum disorders management		Quasi-experimental, 2 arms		3; 64 (NR; NR); 37.9 (8.6); 33%; Patients with schizophrenia spectrum disorder, recruited from hospitals and clinics		78		App		Psychotic symptoms significantly decreased postintervention in the intervention group compared with control (*b=*−0.005; 95% CI −0.01 to −0.0006)		.03	
**Smoking cessation**															
	Businelle, 2016, and Hebert, 2018, United States [37,38]	Smoking cessation and relapse prevention		Quasi-experimental 1 arm		13; 59 (N/A); 52 (7); 54%; Individuals attending a first visit at a smoking cessation clinic		78		App, group counseling, and cessation pharmacotherapy		Abstinence rate decreased over time (41% in week 1 and 20% in week 12)		—	
	Hebert, 2020, United States [39]	Smoking cessation and relapse prevention		Randomized controlled trial, 3 arms		13; 81 (28; 28; 28); 49.6 (11.9); 50%; Individuals referred to a smoking cessation clinic		66		App, group counseling, and cessation pharmacotherapy		Abstinence rate NS between groups		—	
**Substance abuse control**															
	Dulin, 2014, United States [40]	Alcohol abuse treatment		Quasi-experimental 1 arm		5; 28 (N/A); 33.6 (6.5); 46%; Individuals currently in treatment for an alcohol disorder, recruited from the community		100		App and sensor		Decrease in percentage of heavy drinking days postintervention (56% vs 25%; Cohen *d*=1.0)		<.001	
	Leonard, 2017, United States [41]	Alcohol abuse prevention and management		Quasi-experimental, 1 arm		3; 10 (N/A); 20.7 (NR); 100%; College students with problematic drinking not under treatment		100		App, two counseling sessions, and sensor		NR		—	
	Shrier, 2018, United States [42]	Marijuana use cessation		Randomized controlled trial, 3 arms		12; 70 (NR; NR; NR); 20.7 (NR); 60%; Marijuana users from primary care clinics		66		App and counseling sessions		Percentage of days abstinent, NS between arms		—	
**Diet and physical activity**															
	Mundi, 2015, United States [43]		Promote healthy lifestyles to prepare for bariatric surgery		Quasi-experimental, 1 arm		15; 30 (N/A); 41.3 (11.4); 90%; Patients with obesity undergoing assessment for bariatric surgery		67		App		Nutrition knowledge and engagement with healthy lifestyles: NS improvements		—
	Goldstein, 2018 and 2020, United States [44,45]		Diet adherence		Randomized controlled trial, 2 arms		10; 121 (62; 59); 47.2 (13.4); 100%; BMI ≥25 kg/m^2^ recruited from the community		84.3		App and Weight Watchers program		Weight loss: NS improvements; Lapse frequency: NS improvements		—
	Pentikäinen, 2019, Finland [46]		Diet adherence		Quasi-experimental, 1 arm		4; 74 (N/A); 36.2 (12.5); 61%; Individuals interested in well-being, recruited from universities		79		App		The average interval between meals increased; the number of daily eating occasions decreased		.003; .01
	Allicock, 2020, United States [47]		Promote physical activity and diet adherence		Randomized controlled trial, 2 arms		8; 22 (13;9); 52 (9); 100%; African American women post breast cancer treatment, recruited from the community		100		App		Reduced sedentary time by 4.37 (SD 7.14) hours/day versus controls; waist circumference, BMI change, physical activity, diet: NS improvements		<.05

^a^I: intervention.

^b^C: control

^c^N/A: not applicable.

^d^NR: not reported.

^e^Not available.

^f^NS: not supported.

^g^EMA: ecological momentary assessment.

### Intervention Characteristics

The commonly collected types of EMA data were affect-related (eg, emotions, feelings, and mood; 12/17, 71% studies; 14/19, 74% papers) [29-33,35-39,41,42,44,45], related to behaviors (eg, self-reported diet, physical activity, alcohol use, and medication adherence; 12/17, 71% studies; 14/19, 74% papers) [31-39,42-47], related to cognitions (eg, reasons for nonadherence and reasons to quit alcohol; 10/17, 59% studies; 12/19, 63% papers) [29,31,33-39,42-45], and related to social and environmental context (eg, distractions while eating and interaction with someone smoking; 9/17, 53% studies; 11/19, 58% papers; Table 2) [29,36-45]. In addition to user-reported EMAs, 18% (3/17) of studies also had sensor-collected data (eg, GPS and accelerometer) [40,48,49]. User-reported data collection was initiated either by the app (user would be prompted by the app to provide certain data) [29,31,32,34-39,41-45] or by the user (eg, as users saw fit; after a certain event, such as a meal) [29,30,33,37-41,44-46], sometimes with more than one modality in the same study [37-39,41,44,45,47]. The daily frequency of EMA prompts in app-initiated data collection was reported in 11 (65%; 13/19, 68% papers) studies [29,31,32,35-39,42-45,47], ranging from 2-8 times, with the most common being 4 to 5 times daily [29,32,37-39,42,43]. In 12% (2/17) of studies, the daily frequency was variable, depending on the number of times the participant needed to take medication daily [34] and depending on a trigger from a sensor [41]. The time window allowed for responding to EMA prompts was reported in 29% (5/17) of studies and varied between 1 and 130 minutes (Multimedia Appendix 6) [31,36-38,43,44].

EMIs consisted mostly of suggesting coping strategies (eg, use of cognitive-behavioral skills) [29,31-33,35-45,47], followed by motivational feedback (eg, positive reinforcement and supportive messages) [29,32,34,37-43,47] and informational feedback (eg, user-tailored graphs; Table 2) [30,31,44-47]. EMI characteristics were poorly reported and were not described in a standardized manner across studies, rarely detailing the decision mechanism (eg, algorithm). The EMI mechanism was not reported in 7 (41%, 8/19, 42% papers) studies [31-33,35-39], predetermined in 8 (47%) studies [30,34,40-43,46,47], and adaptive in 2 (12%; 3/19, 16% papers) studies [29,44,45]. The delivery format was in the form of text in most studies [29,31-34,36-45,47]; approximately 12% (2/17) of studies used tailored graphs [30,46], and 6% (1/17) of studies used texts and images [35]. Most interventions used other components in addition to the app, the most common one being counseling sessions with a therapist, either face-to-face or by telephone [29,31,34,35,37-39,41,42].

There were 35 BCTs identified across the studies (Multimedia Appendices 7 and 8). The most popular BCTs were social support (unspecified; 13/17, 76% studies; 15/19, 79% papers) [29,31,32,34,35,37-45,47], followed by prompt or cue (10/17, 59% studies; 11/19, 58% papers) [29,30,32,34-36,40,43-45,47], problem solving (9/17, 53% studies; 11/19, 58% papers) [29,31,33,36-41,44,45], feedback on behavior (6/17, 35% studies) [31,34,36,41,43,46], self-monitoring of behavior (7/17, 41% studies) [29,31,34,40,42,46,47], and social support (emotional; 6/17, 35% studies) [31-33,40-42]. The most commonly mentioned theories, frameworks, or models were cognitive behavioral therapy [31,33,41,42] and motivational interviewing [40-42].

**Table 2:** Characteristics of EMA^a^ data collection and EMI^b^ in included studies^c^.

Author, year, location			EMA data collection									EMI				
			Type of user-reported data		Mechanism^d^		Format (input mode)		Sensors^e^		Type of intervention content			Mechanism^d^		Format (delivery mode)
**Mental health**																
	Burns, 2011, United States [29]	Affect-related (mood), cognitions, social and environmental context, and motivational states		App-initiated (predetermined, ≥5 times daily at random times between 7 AM and 10 PM, depending on participant preference) and user-initiated (frequency as users see fit)		Likert scales and multiple choice		38 sensors (eg, GPS and accelerometer)		Coping strategies (suggested activities) and motivational feedback (message to reinforce improvement)			App-initiated and adaptive (eg, suggested activities when a user’s self-reported mood was outside their typical range, based on a machine-learning algorithm built from EMA and sensor data); frequency, interval, and time allowed: NR^f^		Text	
	Bush, 2014, United States [30]	Affect-related and mental health-related symptoms and events (stress, head injury, depression, anxiety, well-being)		User-initiated (frequency as users see fit)		Slide bar to rate emotions and states		—^g^		Informational feedback (access to customized reports of mood data and personalized graphs of EMA data)			App-initiated; predetermined; frequency, interval, and time allowed: N/A^h^		Graph	
	Wenze, 2016, United States [31]	Affect-related, behaviors (daily medications and appointments and adherence behaviors), cognitions (risk factors for nonadherence), and bipolar disorder symptoms (eg, sleep)		App-initiated (time-contingent; 2/day, 9 AM and 9 PM; time allowed: 12 min)		Likert scale and multiple choices		—		Coping strategies and informational feedback			App-initiated; NR		Text	
	Shrier, 2017, United States [32]	Affect-related, behaviors (sexual behavior), and self-efficacy for safer sex behavior		App-initiated (predetermined, at random times, 4 times daily; time allowed: NR)		NR		—		Coping strategies and motivational feedback (provided supportive messages and prompted use of cognitive-behavioral skills)			App-initiated; NR		Text	
	Bakker, 2018, Australia [33]	Affect-related (mood), cognitions, and physiological response		User-initiated (frequency as users see fit)		Multiple choice and sliding bars		—		Coping strategies; upon completion of activities, gamified rewards were issued			NR		Text	
	Kreyenbuhl, 2019, United States [34]	Behaviors (medication adherence at scheduled times throughout the day) and cognitions (reasons for nonadherence)		App-initiated (predetermined, event-contingent and dependent on the number of times the participant needs to take medication daily)		Multiple choice		—		Motivational feedback based on self-reported adherence			App-initiated and predetermined (If-Then, depending on individual responses); frequency and interval dependent on EMA; time allowed: NR		Text	
	Vaessen, 2019, Netherlands [35]	Affect-related (current mood), behaviors (activity), and symptoms		App-initiated (predetermined, random times, 8 times daily); time allowed: NR		NR		—		Coping strategies (suggested exercise to train general acceptance and commitment therapy principles)			App-initiated; NR		Image and text	
	Hanssen, 2020, Netherlands [36]	Affect-related (feelings and moods), thoughts, behaviors, cognitions, social and environmental context, and symptoms		App-initiated (predetermined, random, 6 times daily between 10 AM and 10 PM, intervals >130 min; time allowed: NR)		Likert scale, multiple choices, and yes/no answers		—		Coping strategies (provided suggestions for a certain activity or behavior change based on previous EMA answers in the following categories: psychotic symptoms, social engagement, health behavior, and mood and emotion)			App-initiated; mechanism NR; frequency: 2 prompts/day; interval and time allowed: NR		Text	
**Smoking cessation**																
	Businelle, 2016 [37] and Hebert, 2018, United States [38]	Affect-related, behaviors (recent alcohol consumption), cognitions (motivation to quit), social and environmental context (eg, cigarette availability and interaction with someone smoking), and urge to smokes		Three types of EMA with three different frequencies: Daily diary (app-initiated; once daily, 30 min after waking; time allowed: 60 seconds); Random sampling (app-initiated; predetermined, random, 4 times daily; time allowed: NR); Event sampling (user-initiated; precessation smoking, urge, and postcessation lapse)		Click buttons to report smoking incidents		—		Coping strategies (provided risk-tailored messages to help participants cope with lapse triggers) and motivational feedback			App-initiated; mechanism NR; frequency and interval: NR; time allowed: NR		Text	
	Hebert, 2020, United States [39]	Affect-related, behaviors (recent alcohol consumption), cognitions (motivation to quit), social and environmental context (eg, cigarette availability and interaction with someone smoking), and urge to smoke		Three types of EMA with three different frequencies: Daily diary (app-initiated; 1/day, 30 min after waking); Random sampling (app-initiated; predetermined, random, 4 times daily; time allowed: NR); Event sampling (user-initiated; precessation smoking, urge, and postcessation lapse)		Click buttons to report smoking incidents		—		Coping strategies (provided risk-tailored messages to help participants cope with lapse triggers) and motivational feedback			App-initiated; mechanism NR; frequency and interval: NR; time allowed: NR		Text	
**Substance abuse control**																
	Dulin, 2014, United States [40]	Social and environmental context (user-identified high-risk locations); cravings		User-initiated (frequency as users see fit)		NR		GPS		Coping strategies (provided audible alert and suggestions for maintaining control of drinking when a boundary was crossed around a GPS-triggered high-risk location) and motivational feedback			App-initiated; predetermined; frequency and interval: based on EMA and sensor data; time allowed: NR		Text	
	Leonard, 2017, United States [41]	Affect-related (current emotions and level of intensity) and social and environmental context		App-initiated (event-contingent; frequency and interval: based on trigger from sensor; time allowed: NR) and user-initiated		Multiple choice		Electrodermal activity and accelerometer		Coping strategies (based on cognitive behavioral therapy) and motivational feedback			App-initiated; predetermined; frequency and interval based on EMA and sensor data; time allowed: NR		Text	
	Shrier, 2018, United States [42]	Affect-related, behaviors (use of marijuana), cognitions (personal top three triggers for use and effort to avoid use), social and environmental context, and marijuana desire		App-initiated (random; 4-6 times daily; time allowed: NR)		NR		—		Motivational feedback (provided messages designed to support self-efficacy)			App-initiated; predetermined; frequency and interval based on EMA responses; time allowed: NR		Text	
**Diet and physical activity**																
	Mundi, 2015, United States [43]	Behaviors (frequency of eating or snacking and use of calorie-containing beverages, meal planning, frequency of foods not prepared at home, rate of eating, and quantity of physical activity), cognitions (barriers to physical activity), and social and environmental context (distractions while eating)		App-initiated (predetermined, time-contingent; five times daily; time allowed: 60 min)		NR		—		Coping strategies and motivational feedback; upon a study subject’s response to the given EMA message, a tailored EMI message was electronically generated (if a patient endorsed a healthy lifestyle, they were sent a congratulatory and supportive message, and if a patient was struggling to make a positive lifestyle modification, they were sent a supportive message outlining some alternative behavioral strategies)			App-initiated; predetermined; frequency and interval based on EMA responses; time allowed: NR		Text	
	Goldstein, 2018 and 2020, United States [44,45]	Affect-related, behaviors (dietary lapse), cognitions, and social and environmental context (variables known to predict lapses)		App-initiated (predetermined, six times daily; time allowed: 90 min) and user-initiated (after a lapse)		Likert scales and yes or no answers		—		Coping strategies and informational feedback (alert was issued when the algorithm classified a user to be at risk for lapsing, communicating (a) top three factors contributing to level of risk (context-awareness) and (b) strategies to cope with each specific risk factor)			App-initiated; adaptive; frequency and interval based on EMA responses; time allowed: NR		Text	
	Pentikäinen, 2019, Finland [46]	Behaviors (eating rhythm)		User-initiated (when participant had meal)		Two buttons to record types of eating occasion		—		Informational feedback (graphs of EMA data)			App-initiated; predetermined; frequency and interval based on EMA responses; time allowed: N/A		Tailored graph	
	Allicock, 2020, United States [47]	Behaviors (diet and physical activity)		Three types: Daily diary (app-initiated; 1/day, 30 min after waking; time allowed: NR); Random sampling (app-initiated; predetermined, random, 2 times daily; time allowed: NR); User-initiated (before and after meals or exercise)		NR		—		Informational, coping strategies, and motivational feedback (providing behavioral cues or prompting, increasing self-efficacy, building behavioral capability, and providing positive reinforcements to behaviors)			App-initiated; predetermined; frequency and interval based on EMA responses); time allowed: NR		Text	

^a^EMA: ecological momentary assessment.

^b^EMI: ecological momentary intervention.

^c^EMA and EMI characteristics reported according to items specified in Table 3 based on information reported in the included studies.

^d^Initiative, mechanism, frequency and interval, and time allowed.

^e^Additional components for data collection.

^f^NR: not reported.

^g^Not available.

^h^N/A: not applicable.

### Incentives, Adherence, Reported Outcome Measures, and User Perspectives

Participants in 64% (11/17) studies (13/19, 68% papers) received material (eg, movie tickets) or monetary compensations for participating in the study [31,32,36-42,44-47]. Of those 11 studies, 6 (55%) studies (8/13, 62% papers) had incentives associated with EMA completion [31,37-39,42,44,45,47]. Adherence to EMA prompts (ie, to self-reporting data) was mentioned in 59% (10/17) studies (12/19, 63% papers) [31,34,36-39,41-45,47], most often in the form of response rate (Multimedia Appendix 6). The response rate varied from 30.7%-87% (9/17, 53% studies; 11/19, 58% papers; average 64.7%) [31,34,36-39,42-45,47]. Studies with a time limit to respond to EMA (4/17, 24% studies; 5/19, 26% papers) had lower response rates (30.7%, 58%, 62.9%) [31,43-45], except for 6% (1/17) of studies (2/19, 11% papers), with a response rate of 87% and with a high financial incentive for participants (those who completed 50%-74% of assessments received a US $40 gift card; 75-89% completion, US $80 gift card; and >90%, US $120 gift card) [37,38]. Of the 17 studies, 2 (12%) studies (3/19, 16% papers) reported the average time spent on each EMA prompt (ie, time spent self-reporting data), which varied from 2-6 minutes [31,37,38]. Adherence to EMI was reported in 24% (4/17) of studies (6/19, 32% papers) [36-38,44-46], with different measurements in each study (Multimedia Appendix 6).

Of the 17 studies, health-related outcomes were reported in 12 (71%) studies (14/19, 74% papers) [29,31,34,36-40,42-47]. Of the 17 studies, the 4 (24%) included RCTs reported nonstatistically significant improvements in substance abstinence, diet, weight loss, and sedentary time compared with the control group [39,42,44,47], and only 4 (24%) quasi-experimental studies reported statistically significant pre-post improvements in self-reported primary outcomes, namely depressive (*P*<.001) [29] and psychotic symptoms (*P*=.03) [36], drinking frequency (*P*<.001) [40], and eating patterns (*P*=.01) [46].

Regarding user perspectives (Multimedia Appendix 9), all apps were perceived as useful in supporting behavior change, although to varying degrees. In half of the studies, apps’ ease of use was assessed, with users rating the apps favorably [30-34,36,40-42]. The most helpful aspect of the apps, according to participants, was increasing awareness of their own behavior patterns [29,31-33,36-42,44-46]. The preferred and desirable features of the apps included personalization (eg, tailored prompts, tailored content, and feedback based on user responses) [29,31,32,35-40,44,45], communication with clinicians or coaches [29,30,47], multimedia and interactive capabilities, including voice recording [29,30,32,47], and an appealing design of the graphical user interface [30,32,33,36,40]. Common negative perspectives included EMA prompts being too frequent (more than five times daily), inopportune or tedious to complete [33,35,36,41-45], technical issues (eg, battery drainage and connectivity problems) [29-32,34,40-42], and repetitive content and feedback [31,32,42,44,45]. Of the 17 studies, 2 (12%) studies mentioned the potential negative impacts of momentary prompts on users’ mental well-being, including increased anxiety and stress because of prompts being too frequent or too sudden [41], or prompts giving users an unpleasant degree of self-awareness [35].

### Checklist for Reporting EMA- and EMI-Specific Aspects in Behavior Change Experiments

EMI and EMA components were rarely reported and were not described in a standardized manner across studies. We found that half of the studies failed to report EMA adherence rates, and this was even lower for EMIs. In addition, the mechanism details for EMAs and EMIs and incentives to complete EMAs and adhere to EMIs have been infrequently reported. On the basis of our findings and on an existing CREMAS [22], we developed a set of reporting items to include in the methods and results sections of EMA and EMI experiments (CREMAIs [Checklist for Reporting EMA- and EMI-specific aspects]; Table 3).

**Table 3:** Adapted checklist for reporting smartphone-delivered EMA^a^- and EMI^b^-specific aspects in behavior change experiments (CREMAIs^c^)^d^.

Paper section and item			Description		EMA		EMI
**Methods**							
	Type	Details about the type of EMA and EMI		Type of data collected (eg, affect-related, behaviors, cognitions, and social and environmental context)		Intervention content (eg, coping strategies, motivational feedback, informational feedback, and other behavior change techniques)	
	Mechanism	Initiative		System (eg, app) and/or user-initiated EMA		System (eg, app) and/or user-initiated EMI	
		Mechanism responsible for triggering the EMA/EMI		Predetermined (event-contingent, time-contingent and/or random) or adaptive (eg, using statistical/machine learning methods to adapt EMA prompting based on user data)		Predetermined (eg, IF *X* EMA response, THEN *Y* EMI) or adaptive (eg, using statistical/machine learning methods to adapt EMI based on previous EMA responses and other user data)	
		Frequency and interval		Number of EMA prompts/day and time between each EMA		Number of EMI prompts/day and time between each EMI	
		Time allowed		Total time allowed to answer/receive/perform EMAs before prompt expires		Total time allowed to answer/receive/perform EMIs before prompt expires	
	Format	Details about how EMAs/EMIs are delivered		Input mode (eg, Likert scales, yes/no answers, multiple choice, voice, free-text, and image)		Delivery mode (eg, voice, text, and image)	
	Additional components	Other components used in conjunction with the app (eg, sensors; face-to-face behaviors; and website)		Other components used in conjunction with the app (eg, sensors; face-to-face behaviors; and website)		Other components used in conjunction with the app (eg, sensors; face-to-face behaviors; and website)	
	Behavior change rationale	Theories/frameworks/models to inform the design of the intervention		Theories/frameworks/models to inform the design of the intervention		Theories/frameworks/models to inform the design of the intervention	
	Incentives	Incentives provided for EMA/EMI adherence		Incentives provided for EMA adherence		Incentives provided for EMI adherence	
**Results**							
	Response latency	Average time to respond to EMA/EMI prompt		Average time to respond to EMA prompt		Average time to respond to EMI prompt	
	Time spent per prompt	Average time spent per EMA/EMI prompt		Average time spent per EMA prompt		Average time spent per EMI prompt	
	Adherence rate	Response or adherence rate for EMA/EMI prompts, detailing the total number of prompts answered/EMI suggestions implemented, and the total number of prompts delivered		Response or adherence rate for EMA prompts, detailing the total number of prompts answered/EMI suggestions implemented, and the total number of prompts delivered		Response or adherence rate for EMI prompts, detailing the total number of prompts answered/EMI suggestions implemented, and the total number of prompts delivered	
	Missing data	Report whether EMA/EMI adherence is related to demographic or other variables (eg, prompt relevance)		Report whether EMA adherence is related to demographic or other variables (eg, prompt relevance)		Report whether EMI adherence is related to demographic or other variables (eg, prompt relevance)	

^a^EMA: ecological momentary assessment.

^b^EMI: ecological momentary intervention.

^c^CREMAIs: checklist for reporting EMA and EMI-specific aspects.

^d^Adapted from Liao et al [22].

## Discussion

### Principal Findings

Although the potential for EMIs that build on EMA data for behavior change in the smartphone era seems promising, research on this approach is lacking. We identified 17 studies (only 4 RCTs), all with small sample sizes, short follow-up, and limited evaluation of efficacy. EMIs described were predominantly in mental health management, with a few addressing smoking cessation, substance abuse, diet, weight loss, and physical activity. The most common type of EMA data collected were related to subjective experiences, namely affective states and cognitions, indicating the usefulness of EMAs for this purpose. Behaviors were also often collected via EMAs, with sensors rarely being used. Adherence to collection of EMA data was a common barrier to implementation, with participants disliking the high frequency and tedious nature of EMA data collection. This suggests that EMAs could be gathered via other methods preferred by users (eg, voice). In addition, EMAs could be coupled with passively collected sensor data whenever possible to decrease user burden while still enabling the collection of subjective experiences relevant to user-desired personalization.

Description of interventions and reporting of evaluation measures were heterogeneous in each health domain, and there were few studies per health domain, limiting any conclusion being made on their efficacy on health behaviors, engagement, and outcomes.

### Comparison With Existing Literature

To our knowledge, this is the first systematic review of smartphone-delivered EMIs based on self-reported EMAs to support behavioral changes. Existing reviews of EMIs in the treatment of psychotic disorders [24], major depressive disorder [50], alcohol use [51], and eating disorders [14] found that most interventions were in the early stages of development, which aligns with the findings of this review. Notably, the present findings show that most uses of EMIs based on EMAs to date seem to be in the field of mental health, where emotional and cognitive states can vary considerably throughout the day and influence behaviors. Previous systematic reviews on EMIs have all focused on mental health, used mostly older technologies, and did not tailor EMIs based on EMAs, having found mixed results (2 meta-analyses [23,25] showing small but positive effect sizes and another systematic review demonstrating acceptability and feasibility [24]).

Our review found that EMI and EMA components were rarely reported and were not described in a standardized manner across studies, hampering progress in this field. EMA- and EMI-specific aspects, such as the triggering mechanism and incentives, are important determinants of intervention uptake, retention, and efficacy. Hence, this poor reporting makes it difficult to synthesize and replicate existing evidence. Thus, we developed a set of reporting items—a checklist for reporting EMA- and EMI-specific aspects in behavior change experiments (CREMAIs)—based on an existing reporting checklist for EMA studies (CREMAS) [22]. Given that our adapted checklist focuses exclusively on EMA and EMI aspects, it should be used in conjunction with other reporting guidelines, depending on the type of experimental study design [52-55]. Our findings extend on previous systematic reviews in the field and add to the CREMAS checklist [22] by providing a detailed description of both EMI and EMA components (not just EMA) and specifically with respect to interventions that use smartphones.

EMI users had negative feedback regarding technical issues, inopportune and repetitive alerts, and prompts not being tailored enough, which may decrease participant engagement. The most common recommendations for intervention design were to make the intervention more personalized and engaging (eg, personalized coping strategies) and to tailor data collection and reduce reporting burden and invasiveness. These perspectives expand on existing literature by showing that for sustained efficacy of behavior change interventions, user engagement is paramount [4,6,56]. Personalization has been commonly suggested as a way to make interventions more engaging, effective, and better received by users [57-60]. One example includes *just-in-time adaptive interventions*, which are system-triggered interventions that aim to provide the right type/amount of support, at the right time, by adapting to an individual’s changing internal and contextual state (usually based on sensor-collected data) [61].

### Strengths and Limitations

This review has several strengths. We developed and followed a protocol that was registered in the PROSPERO database at the start of the study. Intervention components were characterized in detail, including the coding of BCTs. However, the results of this review need to be interpreted in the context of certain limitations. Owing to the small number of RCTs, a meta-analysis was not conducted, and thus it was not possible to provide an estimation of preliminary efficacy. There was low to moderate agreement in screening, which reflects the difficulty in establishing whether a study met the inclusion criteria. Screening was complicated by incomplete intervention descriptions, particularly with regard to EMI and EMA reporting. Finally, the definitions of EMI and EMA are not consensual in the literature. Thus, the studies included in this review reflect the predefined definitions we adopted.

### Implications for Future Studies

The use of smartphone-delivered EMIs based on EMAs in behavior change interventions is a novel area of research, where more RCTs are needed to determine efficacy. Given the ubiquity of smartphones, these interventions have the potential to support behavioral changes at scale. Nevertheless, it is still uncertain which populations may find the use of EMIs based on EMAs most acceptable and which populations and settings may benefit the most. So far, studies have focused on mental health, smoking, substance abuse, diet, weight loss, and physical activity, with mixed results. Appropriately powered clinical trials are needed to examine the use of EMIs tailored by EMAs in a range of populations and settings and to examine the impact on health outcomes and the longevity of these benefits.

Future studies should explore the combination of EMAs and sensor data to deliver more personalized and minimally burdensome EMIs. EMA involves manual data collection at several points in time, which can be burdensome for users, but remains important to gather individual data that sensors are currently unable to capture, such as subjectively perceived cognitive and affective states [62]. Capturing subjective experiences (eg, cravings, pain, and loneliness) enables a richer and deeper insight into a person’s behavior and can foster the tailoring of an intervention to a person’s needs, which in turn may increase the perceived relevance of EMIs. By combining self-reported EMAs of subjective experiences with additional objective data passively collected via sensors (eg, physical activity patterns and heart rate) [63,64], there is potential to promote a more engaging personalized intervention, as minimally burdensome as possible. Novel machine learning algorithms can further explore these different types of data to increase the precision of personalized interventions [65].

A more seamless EMA and EMI experience is crucial for engagement. User burden associated with data entry is the most reported reason why people stop using mobile health apps [66]. In addition to using sensors whenever possible, another possibility to reduce user burden is to optimize the design of data collection modes. For instance, faster methods, such as speech-based data entry, may be used instead of requiring users to type in response [67]. Another option would be the use of a chatbot to enable data collection in a conversational and more engaging way. Other feasible options include data entry templates, such as dropdown menus, and the use of personalization to autopopulate some data fields [68] based on previous entries or other data sources [33]. Co-designing interventions with users may offer insights into the best options for data collection in each particular case, regarding the types and amount of data, and the mode, frequency, and timing of data collection [69].

Future research in this area should adhere to existing reporting standards, namely, what concerns the detailing of EMA- and EMI-specific characteristics. Reporting guidelines are essential in facilitating the evaluation of study validity and allowing for comparisons across interventions. Consistency and detail in reporting intervention characteristics enable replication efforts and allow for meta-analyses and meta-regression to explore the features associated with the highest user engagement and intervention efficacy. Advancements in the field of EMAs and EMIs and the higher scientific impact of published studies in this area are dependent on the consistent use of reporting guidelines.

### Conclusions

This is the first systematic review of smartphone-delivered EMIs based on self-reported EMAs promoting health behaviors. The use of this approach in behavior change is an emerging area of research, with few studies evaluating efficacy and most interventions focusing on mental health management. EMAs were commonly used to capture subjective experiences, as well as behaviors, whereas sensors were rarely used. Future research should explore combining self-reported EMAs of subjective experiences with objective data passively collected via sensors to promote personalization. Studies should also explore the effects of different EMA data collection methods (eg, chatbots) on user burden, engagement, and efficacy. A reporting checklist was developed with the goal of facilitating interpretation and comparison of findings and enhancing transparency and replicability in future studies using EMAs and EMIs.

## Appendix

Multimedia Appendix 1 Search strings.

Multimedia Appendix 2 Inclusion and exclusion criteria.

Multimedia Appendix 3 List of articles excluded after full-text review for not meeting inclusion criteria regarding the population, intervention, outcome or study design.

Multimedia Appendix 4 Risk of bias of included randomized controlled trial.

Multimedia Appendix 5 Risk of bias of nonrandomized studies.

Multimedia Appendix 6 Ecological momentary assessment adherence, ecological momentary intervention adherence, and incentives used.

Multimedia Appendix 7 Behavior change techniques present in the interventions of included studies.

Multimedia Appendix 8 Behaviour change techniques used in each study and excerpts.

Multimedia Appendix 9 User perspectives and suggestions.

## Abbreviations

BCT: behavior change technique
CREMAIs: checklist for reporting ecological momentary assessment and ecological momentary intervention-specific aspects
CREMAS: checklist for reporting ecological momentary assessment studies
EMA: ecological momentary assessment
EMI: ecological momentary intervention
PICO: Participants, Intervention, Comparator, Outcomes
PRISMA: Preferred Reporting Items for Systematic Reviews and Meta-Analyses
PROSPERO: International Prospective Register of Systematic Reviews
RCT: randomized controlled trial
