# Supplementary material for: Smartphone-Delivered Ecological Momentary Interventions Based on Ecological Momentary Assessments to Promote Health Behaviors: Systematic Review and Adapted Checklist for Reporting Ecological Momentary Assessment and Intervention Studies
Source: JMIR Mhealth Uhealth. 2021 Nov 19;9(11):e22890. doi: 10.2196/22890 (PMC8663593; doi:10.2196/22890)
Supplement: Multimedia Appendix 1 [file mhealth_v9i11e22890_app1.docx]

# **Multimedia Appendix 1: Search strings**

1. **Pubmed:**

#1 ("ecological momentary assessment"[MeSH Terms] OR ("ecological"[All Fields] AND "momentary"[All Fields] AND "assessment"[All Fields]) OR "ecological momentary assessment"[All Fields] OR (ambulatory[All Fields] AND "assessment"[All Fields]) OR "ambulatory assessment"[All fields] OR "ecological momentary"[All Fields] OR "experience sampling"[All Fields])

**AND**

#2 ("smartphone"[MeSH Terms] OR "smartphone"[All Fields] OR smartphone*[All Fields] OR "smart phone"[All Fields] OR "smart phones"[All Fields] OR "mobile health"[All Fields] OR "mobile applications"[All Fields] OR "mobile applications"[MeSH Terms] OR "mobile application"[All Fields] OR "telemedicine"[MeSH Terms] OR "telemedicine"[All Fields] OR "mhealth"[All Fields] OR wearable[All Fields] OR "fitness trackers"[All Fields] OR "fitness trackers"[MeSH Terms] OR "fitness tracker"[All Fields] OR "cell phone"[All Fields] OR "cell phones"[All Fields] OR "cell phone"[MeSH Terms] OR "cellphone"[All Fields] OR cellphone's[All Fields] OR cellphones[All Fields] OR "cellular phone"[All Fields] OR "cellular phones"[All Fields])

**Results: 1137**

1. **Embase**

#1 (ecological momentary assessment OR ecological momentary assessment / OR ambulatory assessment OR ecological momentary OR experience sampling)

**AND**

#2 (smartphone* OR smart phone OR smart phones OR mobile health OR mobile phone/ or smartphone/ OR mobile applications OR mobile application OR mhealth OR telemedicine OR wearable or fitness trackers OR fitness tracker OR activity tracker/ OR cell phone OR cell phones OR cellphone* OR cellular phone OR cellular phones).mp. [mp=title, abstract, heading word, drug trade name, original title, device manufacturer, drug manufacturer, device trade name, keyword, floating subheading word, candidate term word]

**Results: 599**

1. **PsycINFO**

#1 (ecological momentary assessment OR ambulatory assessment OR ecological momentary OR experience sampling)

**AND**

#2 (smartphone* OR smart phone OR smart phones OR exp Mobile Devices/ OR activity tracker OR activity trackers OR mobile health OR mobile applications OR mobile application OR mhealth OR telemedicine OR wearable OR fitness trackers OR fitness tracker OR cell phone OR cell phones OR cellphone* OR cellular phone OR Cellular Phone/ OR cellular phones). mp. [mp=title, abstract, heading word, table of contents, key concepts, original title, tests & measures]
**Results: 395**

1. **CINAHL complete (SmartText mode)**

#1 (ecological momentary assessment OR ambulatory assessment OR ecological momentary OR experience sampling)

**AND**

#2 (smartphone* OR smart phone OR smart phones OR mobile health OR mobile applications OR mobile application OR mhealth OR wearable OR fitness trackers OR fitness tracker OR activity tracker OR activity trackers OR cell phone OR cell phones OR cellphone* OR cellular phone OR cellular phones)).

**Results: 690**
