# Supplementary material for: Smartphone-Delivered Ecological Momentary Interventions Based on Ecological Momentary Assessments to Promote Health Behaviors: Systematic Review and Adapted Checklist for Reporting Ecological Momentary Assessment and Intervention Studies
Source: JMIR Mhealth Uhealth. 2021 Nov 19;9(11):e22890. doi: 10.2196/22890 (PMC8663593; doi:10.2196/22890)
Supplement: Multimedia Appendix 2 [file mhealth_v9i11e22890_app2.docx]

# **Multimedia Appendix 2: Inclusion/ Exclusion criteria**

|  | **Inclusion** | **Exclusion** |
| --- | --- | --- |
| Population | Adults 18 years of age or older (e.g chronic disease patients, healthy people, mental health patients…) |  |
| Intervention | - Delivery of an intervention (EMI) provided to people during their everyday lives (i.e. in real time) and settings (i.e. real world) via a smartphone, based on EMA (i.e. repeated reports by a research participant of symptoms, affect, behavior, or cognition close in time to experience and in the participant's natural environment.).  - Both EMA and EMI need to occur via smartphone. | - Intervention only based on passive data collection (e.g. sensors)  - SMS only, PDA, web-based, tablet, email  - Studies that only use EMA as a method of data collection/to measure outcomes |
| Comparison | any |  |
| Outcome | • any health-related measures  • effects of EMI • perceptions about EMI • engagement with EMI |  |
| Study type | Experimental studies | • protocols • reviews  • opinion pieces  • design  • development papers |

Abbreviations: EMA, ecological momentary assessment; EMI, ecological momentary intervention
