# Supplementary material for: Smartphone-Delivered Ecological Momentary Interventions Based on Ecological Momentary Assessments to Promote Health Behaviors: Systematic Review and Adapted Checklist for Reporting Ecological Momentary Assessment and Intervention Studies
Source: JMIR Mhealth Uhealth. 2021 Nov 19;9(11):e22890. doi: 10.2196/22890 (PMC8663593; doi:10.2196/22890)
Supplement: Multimedia Appendix 3 [file mhealth_v9i11e22890_app3.docx]

# **Multimedia Appendix 3: List of articles excluded after full-text review for not meeting inclusion criteria regarding the population, intervention, outcome or study design**

Intervention:

5. Han H, Zhang J, Hser Y, Liang D, Li X, Wang S et al. Feasibility of a Mobile Phone App to Support Recovery From Addiction in China: Secondary Analysis of a Pilot Study. JMIR mHealth and uHealth. 2018;6(2):e46.

Amorim A, Pappas E, Simic M, Ferreira M, Jennings M, Tiedemann A et al. Integrating Mobile-health, health coaching, and physical activity to reduce the burden of chronic low back pain trial (IMPACT): a pilot randomised controlled trial. BMC Musculoskeletal Disorders. 2019;20(1).

Batink T, Bakker J, Vaessen T, Kasanova Z, Collip D, van Os J et al. Acceptance and Commitment Therapy in Daily Life Training: A Feasibility Study of an mHealth Intervention. JMIR mHealth and uHealth. 2016;4(3):e103.

Brookie K, Mainvil L, Carr A, Vissers M, Conner T. The development and effectiveness of an ecological momentary intervention to increase daily fruit and vegetable consumption in low-consuming young adults. Appetite. 2017;108:32-41.

Burnett-Zeigler I, Waldron E, Hong S, Yang A, Wisner K, Ciolino J. Accessibility and feasibility of using technology to support mindfulness practice, reduce stress and promote long term mental health. Complementary Therapies in Clinical Practice. 2018;33:93-99.

Cadmus-Bertram L, Marcus B, Patterson R, Parker B, Morey B. Randomized Trial of a Fitbit-Based Physical Activity Intervention for Women. American Journal of Preventive Medicine. 2015;49(3):414-418.

Daugherty D, Runyan J, Steenbergh T, Fratzke B, Fry B, Westra E. Smartphone delivery of a hope intervention: Another way to flourish. PLOS ONE. 2018;13(6):e0197930.

den Bakker C, Huirne J, Schaafsma F, de Geus C, Bonjer H, Anema J. Electronic Health Program to Empower Patients in Returning to Normal Activities After Colorectal Surgical Procedures: Mixed-Methods Process Evaluation Alongside a Randomized Controlled Trial. Journal of Medical Internet Research. 2019;21(1):e10674.

Dennis M, Scott C, Funk R, Nicholson L. A Pilot Study to Examine the Feasibility and Potential Effectiveness of Using Smartphones to Provide Recovery Support for Adolescents. Substance Abuse. 2014;36(4):486-492.

Depp C, Ceglowski J, Wang V, Yaghouti F, Mausbach B, Thompson W et al. Augmenting psychoeducation with a mobile intervention for bipolar disorder: A randomized controlled trial. Journal of Affective Disorders. 2015;174:23-30.

Depp C, Kim D, Vergel de Dios L, Wang V, Ceglowski J. A Pilot Study of Mood Ratings Captured by Mobile Phone Versus Paper-and-Pencil Mood Charts in Bipolar Disorder. Journal of Dual Diagnosis. 2012;8(4):326-332.

Duscha B, Piner L, Patel M, Craig K, Brady M, McGarrah R et al. Effects of a 12-week mHealth program on peak VO2 and physical activity patterns after completing cardiac rehabilitation: A randomized controlled trial. American Heart Journal. 2018;199:105-114.

Ellis T, Cavanaugh J, DeAngelis T, Hendron K, Thomas C, Saint-Hilaire M et al. Comparative Effectiveness of mHealth-Supported Exercise Compared With Exercise Alone for People With Parkinson Disease: Randomized Controlled Pilot Study. Physical Therapy. 2019;99(2):203-216.

Everhart R, Heron K, Leibach G, Miadich S. Developing a Mobile Health Intervention for Low-Income, Urban Caregivers of Children with Asthma: A Pilot Study. Pediatric Allergy, Immunology, and Pulmonology. 2017;30(4):252-256.

Fishbach A, Hofmann W. Nudging self-control: A smartphone intervention of temptation anticipation and goal resolution improves everyday goal progress. Motivation Science. 2015;1(3):137-150.

Garcia-Palacios A, Herrero R, Belmonte M, Castilla D, Guixeres J, Molinari G et al. Ecological momentary assessment for chronic pain in fibromyalgia using a smartphone: A randomized crossover study. European Journal of Pain. 2013;18(6):862-872.

Garrison K, Pal P, O’Malley S, Pittman B, Gueorguieva R, Rojiani R et al. Craving to Quit: A Randomized Controlled Trial of Smartphone App–Based Mindfulness Training for Smoking Cessation. Nicotine & Tobacco Research. 2018;.

Gibson K. Efficacy of a self-monitoring intervention for college students with attention problems. [Ph.D]. University of California Los Angeles; 2016.

Gilson N, Pavey T, Vandelanotte C, Duncan M, Gomersall S, Trost S et al. Chronic disease risks and use of a smartphone application during a physical activity and dietary intervention in Australian truck drivers. 2016.

Gilson N, Pavey T, Wright O, Vandelanotte C, Duncan M, Gomersall S et al. The impact of an m-Health financial incentives program on the physical activity and diet of Australian truck drivers. BMC Public Health. 2017;17(1).

Hantsoo L, Criniti S, Khan A, Moseley M, Kincler N, Faherty L et al. A Mobile Application for Monitoring and Management of Depressed Mood in a Vulnerable Pregnant Population. Psychiatric Services. 2018;69(1):104-107.

Hareva D, Okada H, Kitawaki H, Oka H. Supportive intervention using a mobile phone in behavior modification. Acta Med Okayama. 2009;.

Harris B, Melton B, Bland H, Carpentier A, Gonzales J, Catenacci K. Enhancing Psychosocial Constructs Associated with Technology-Based Physical Activity: A Randomized Trial Among African American Women. American Journal of Health Education. 2018;49(2):74-85.

Hooke M, Gilchrist L, Tanner L, Hart N, Withycombe J. Use of a Fitness Tracker to Promote Physical Activity in Children With Acute Lymphoblastic Leukemia. Pediatric Blood & Cancer. 2016;63(4):684-689.

Hutcheson T. Using mobile technology to impact fruit and vegetable consumption in low-income youth. 2013;.

Kazemi D, Borsari B, Levine M, Shehab M, Nelson M, Dooley B et al. Real-time demonstration of a mHealth app designed to reduce college students hazardous drinking. Psychological Services. 2019;16(2):255-259.

Koleilat M, Kim L, Whaley S. Focusing on Excessive Gestational Weight Gain through Weight Tracking Among Participants of the Special Supplemental Nutrition Program for Women, Infants, and Children (WIC) in Southern California. Californian Journal of Health Promotion. 2017;15(3):15-24.

Kooiman T, de Groot M, Hoogenberg K, Krijnen W, van der Schans C, Kooy A. Self-tracking of Physical Activity in People With Type 2 Diabetes. CIN: Computers, Informatics, Nursing. 2018;36(7):340-349.

Koontz B, Sutton L, Levine E, Li X, McSherry F, Peterson B et al. Encouraging Exercise Through Wearable Activity Tracker With Daily Text Feedback: Lessons Learned in Developing the ICanSTEP Program. International Journal of Radiation Oncology*Biology*Physics. 2017;99(2):E539.

LaFreniere L, Newman M. A BRIEF ECOLOGICAL MOMENTARY INTERVENTION FOR GENERALIZED ANXIETY DISORDER: A RANDOMIZED CONTROLLED TRIAL OF THEWORRY OUTCOME JOURNAL. Depression and Anxiety. 2016;33(9):829-839.

Le A, Mitchell H, Zheng D, Rotatori J, Fahey J, Ness K et al. A home-based physical activity intervention using activity trackers in survivors of childhood cancer: A pilot study. Pediatric Blood & Cancer. 2016;64(2):387-394.

Levin M, Haeger J, Cruz R. Tailoring Acceptance and Commitment Therapy Skill Coaching in the Moment Through Smartphones: Results from a Randomized Controlled Trial. Mindfulness. 2018;10(4):689-699.

Levinson C, Fewell L, Brosof L. My Fitness Pal calorie tracker usage in the eating disorders. Eating Behaviors. 2017;27:14-16.

Lindsay E, Chin B, Greco C, Young S, Brown K, Wright A et al. How mindfulness training promotes positive emotions: Dismantling acceptance skills training in two randomized controlled trials. Journal of Personality and Social Psychology. 2018;115(6):944-973.

Lindsay E, Young S, Brown K, Smyth J, Creswell J. Mindfulness training reduces loneliness and increases social contact in a randomized controlled trial. Proceedings of the National Academy of Sciences. 2019;116(9):3488-3493.

Macias C, Panch T, Hicks Y, Scolnick J, Weene D, Öngür D et al. Using Smartphone Apps to Promote Psychiatric and Physical Well-Being. Psychiatric Quarterly. 2015;86(4):505-519.

Mason A, Jhaveri K, Cohn M, Brewer J. Testing a mobile mindful eating intervention targeting craving-related eating: feasibility and proof of concept. Journal of Behavioral Medicine. 2017;41(2):160-173.

Miller C, Weinhold K, Mitchell D. Using Ecological Momentary Assessment to Track Goal Progress Toward the Adoption of a Low Glycemic Index Diet Among Adults With Type 2 Diabetes. Topics in Clinical Nutrition. 2016;31(4):323-334.

Minami H, Brinkman H, Nahvi S, Arnsten J, Rivera-Mindt M, Wetter D et al. Rationale, design and pilot feasibility results of a smartphone-assisted, mindfulness-based intervention for smokers with mood disorders: Project mSMART MIND. Contemporary Clinical Trials. 2018;66:36-44.

Morris M, Kathawala Q, Leen T, Gorenstein E, Guilak F, Labhard M et al. Mobile Therapy: Case Study Evaluations of a Cell Phone Application for Emotional Self-Awareness. Journal of Medical Internet Research. 2010;12(2):e10.

Morrison L, Hargood C, Lin S, Dennison L, Joseph J, Hughes S et al. Understanding Usage of a Hybrid Website and Smartphone App for Weight Management: A Mixed-Methods Study. Journal of Medical Internet Research. 2014;16(10):e201.

O'Brien T, Hathaway D, Russell C, Moore S. Merging an Activity Tracker with SystemCHANGE™ to Improve Physical Activity in Older Kidney Transplant Recipients. Nephrol Nurs J. 2017;.

Olsen H, Brown W, Kolbe-Alexander T, Burton N. A Brief Self-Directed Intervention to Reduce Office Employees’ Sedentary Behavior in a Flexible Workplace. Journal of Occupational and Environmental Medicine. 2018;60(10):954-959.

Olson C, Groth S, Graham M, Reschke J, Strawderman M, Fernandez I. The effectiveness of an online intervention in preventing excessive gestational weight gain: the e-moms roc randomized controlled trial. BMC Pregnancy and Childbirth. 2018;18(1).

Ong S, Jassal S, Miller J, Porter E, Cafazzo J, Seto E et al. Integrating a Smartphone–Based Self–Management System into Usual Care of Advanced CKD. Clinical Journal of the American Society of Nephrology. 2016;11(6):1054-1062.

Pramana G, Parmanto B, Kendall P, Silk J. The SmartCAT: An m-Health Platform for Ecological Momentary Intervention in Child Anxiety Treatment. Telemedicine and e-Health. 2014;20(5):419-427.

Pramana G, Parmanto B, Lomas J, Lindhiem O, Kendall P, Silk J. Using Mobile Health Gamification to Facilitate Cognitive Behavioral Therapy Skills Practice in Child Anxiety Treatment: Open Clinical Trial. JMIR Serious Games. 2018;6(2):e9.

Ryan P, Papanek P, Csuka M, Brown M, Hopkins S, Lynch S et al. Background and method of the Striving to be Strong study a RCT testing the efficacy of a m-health self-management intervention. Contemporary Clinical Trials. 2018;71:80-87.

Ryu B, Kim N, Heo E, Yoo S, Lee K, Hwang H et al. Impact of an Electronic Health Record-Integrated Personal Health Record on Patient Participation in Health Care: Development and Randomized Controlled Trial of MyHealthKeeper. Journal of Medical Internet Research. 2017;19(12):e401.

Shin D, Yun J, Shin J, Kwon H, Min H, Joh H et al. Enhancing physical activity and reducing obesity through smartcare and financial incentives: A pilot randomized trial. Obesity. 2017;25(2):302-310.

Smith J, Whisenhunt B, Buchanan E, Hudson D. Evaluating the effectiveness of ecological momentary assessment and intervention targeting body checking behaviors. Eating Disorders. 2019;27(6):521-537.

Smith W, Zucker-Levin A, Mihalko W, Williams M, Loftin M, Gurney J. A Randomized Study of Exercise and Fitness Trackers in Obese Patients After Total Knee Arthroplasty. Orthopedic Clinics of North America. 2019;50(1):35-45.

Soares Teles A, Rocha A, José da Silva e Silva F, Correia Lopes J, O’Sullivan D, Van de Ven P et al. Enriching Mental Health Mobile Assessment and Intervention with Situation Awareness. Sensors. 2017;17(12):127.

Steinert A, Haesner M, Tetley A, Steinhagen-Thiessen E. Self-Monitoring of Health-Related Goals in Older Adults with Use of a Smartphone Application. Activities, Adaptation & Aging. 2016;40(2):81-92.

Svagård I, Austad H, Seeberg T, Vedum J, Liverud A, Mathiesen B et al. A usability study of a mobile monitoring system for congestive heart failure patients. 2014;.

Swendeman D, Comulada W, Koussa M, Worthman C, Estrin D, Rotheram-Borus M et al. Longitudinal Validity and Reliability of Brief Smartphone Self-Monitoring of Diet, Stress, and Physical Activity in a Diverse Sample of Mothers. JMIR mHealth and uHealth. 2018;6(9):e176.

Tong H, Coiera E, Laranjo L. Using a Mobile Social Networking App to Promote Physical Activity: A Qualitative Study of Users’ Perspectives. Journal of Medical Internet Research. 2018;20(12):e11439.

Valentiner L, Thorsen I, Kongstad M, Brinkløv C, Larsen R, Karstoft K et al. Effect of ecological momentary assessment, goal-setting and personalized phone-calls on adherence to interval walking training using the InterWalk application among patients with type 2 diabetes—A pilot randomized controlled trial. PLOS ONE. 2019;14(1):e0208181.

Valle C, Deal A, Tate D. Preventing weight gain in African American breast cancer survivors using smart scales and activity trackers: a randomized controlled pilot study. Journal of Cancer Survivorship. 2016;11(1):133-148.

van der Meij E, Huirne J, ten Cate A, Stockmann H, Scholten P, Davids P et al. A Perioperative eHealth Program to Enhance Postoperative Recovery After Abdominal Surgery: Process Evaluation of a Randomized Controlled Trial. Journal of Medical Internet Research. 2018;20(1):e1.

Van der Walt N, Salmon L, Gooden B, Lyons M, O'Sullivan M, Martina K et al. Feedback From Activity Trackers Improves Daily Step Count After Knee and Hip Arthroplasty: A Randomized Controlled Trial. The Journal of Arthroplasty. 2018;33(11):3422-3428.

van Os J, Verhagen S, Marsman A, Peeters F, Bak M, Marcelis M et al. The experience sampling method as an mHealth tool to support self-monitoring, self-insight, and personalized health care in clinical practice. Depression and Anxiety. 2017;34(6):481-493.

van Os J, Verhagen S, Marsman A, Peeters F, Bak M, Marcelis M et al. The experience sampling method as an mHealth tool to support self-monitoring, self-insight, and personalized health care in clinical practice. Depression and Anxiety. 2017;34(6):481-493.

Woess S, Baumgartner C, Fetz B, van der Heidt A, Kastner P, Modre-Osprian R et al. Evaluation of an Integrated Telemonitoring Surveillance System in Patients with Coronary Heart Disease. Methods of Information in Medicine. 2015;54(05):388-397.

Wright C, Dietze P, Agius P, Kuntsche E, Livingston M, Black O et al. Mobile Phone-Based Ecological Momentary Intervention to Reduce Young Adults’ Alcohol Use in the Event: A Three-Armed Randomized Controlled Trial. JMIR mHealth and uHealth. 2018;6(7):e149.
