# Supplementary material for: Smartphone-Delivered Ecological Momentary Interventions Based on Ecological Momentary Assessments to Promote Health Behaviors: Systematic Review and Adapted Checklist for Reporting Ecological Momentary Assessment and Intervention Studies
Source: JMIR Mhealth Uhealth. 2021 Nov 19;9(11):e22890. doi: 10.2196/22890 (PMC8663593; doi:10.2196/22890)
Supplement: Multimedia Appendix 4 [file mhealth_v9i11e22890_app4.docx]

# **Multimedia Appendix 4: Risk of bias of included RCTs**

| **Author, year** | **Random sequence allocation** | **Allocation concealment** | **Blinding of participants and personnel** | **Blinding of outcome assessment** | **Incomplete outcome data** | **Selective reporting** |
| --- | --- | --- | --- | --- | --- | --- |
| Hebert, 2020 | Low | Unclear | High | Unclear | Low | Low |
| Shrier, 2018 | Unclear | Unclear | High | High | Low | Unclear |
| Goldstein, 2018, 2020 | Unclear | Unclear | High | Low | Low | Unclear |
| Allicock, 2020 | Unclear | Unclear | High | Low | Low | Unclear |

Legend: Low risk of bias; High risk of bias; Unclear risk of bias
