# Supplementary material for: Smartphone-Delivered Ecological Momentary Interventions Based on Ecological Momentary Assessments to Promote Health Behaviors: Systematic Review and Adapted Checklist for Reporting Ecological Momentary Assessment and Intervention Studies
Source: JMIR Mhealth Uhealth. 2021 Nov 19;9(11):e22890. doi: 10.2196/22890 (PMC8663593; doi:10.2196/22890)
Supplement: Multimedia Appendix 5 [file mhealth_v9i11e22890_app5.docx]

# **Multimedia Appendix 5: Risk of bias of non-randomised studies**

| **Assessment of risk of bias using the Risk Of Bias In Non-randomized Studies – of Interventions (ROBINS-I) assessment tool** | | | | | | | | |
| --- | --- | --- | --- | --- | --- | --- | --- | --- |
| **Risk of bias pre-intervention and at-intervention domains** | | | | **Risk of bias post-intervention domains** | | | |  |
| **Study** | **Bias due to confounding** | **Bias in selection of participants into the study** | **Bias in classification of interventions** | **Bias due to deviations from intended intervention** | **Bias due to missing data** | **Bias in measurement of outcomes** | **Bias in selection of the reported result** | **Overall Assessment of bias** |
| **Mental health** | | | | | | | | |
| Burns, 2011, | Critical | Moderate | Low | No information | Low | Serious | No information | Serious |
| Bush, 2014 | Critical | Moderate | Low | No information | Low | Moderate | No information | Serious |
| Wenze, 2016 | Critical | Moderate | Low | Low | Low | Moderate | No information | Serious |
| Shrier, 2017 | Critical | Moderate | Low | No information | No information | Moderate | No information | Serious |
| Bakker, 2018 | Critical | Moderate | Low | No information | No information | Moderate | No information | Serious |
| Kreyenbuhl, 2019 | Critical | Moderate | Low | Low | Low | Serious | No information | Serious |
| Vaessen, 2019 | Critical | Moderate | Low | No information | No information | Moderate | No information | Serious |
| Hanssen, 2020 | Serious | Low | Low | Low | Low | Serious | No information | Moderate |
| **Smoking cessation** | | | | | | | | |
| Businelle, 2016, and Hebert, 2018 | Critical | Low | Low | Low | Low | Moderate | No information | Moderate |
| **Substance abuse control** | | | | | | | | |
| Dulin, 2014 | Critical | Low | Low | No information | Low | Serious | No information | Serious |
| Leonard, 2017 | Critical | Moderate | Low | No information | Low | Moderate | No information | Serious |
| **Diet and physical activity** | | | | | | | | |
| Mundi, 2015 | Critical | Low | Low | Moderate | Low | Moderate | No information | Moderate |
| Pentikäinen, 2019 | Critical | Low | Low | No information | Low | Serious | No information | Serious |

Key: Low risk of bias, Moderate risk of bias, Serious risk of bias, Critical risk of bias, No information
