# Supplementary material for: Smartphone-Delivered Ecological Momentary Interventions Based on Ecological Momentary Assessments to Promote Health Behaviors: Systematic Review and Adapted Checklist for Reporting Ecological Momentary Assessment and Intervention Studies
Source: JMIR Mhealth Uhealth. 2021 Nov 19;9(11):e22890. doi: 10.2196/22890 (PMC8663593; doi:10.2196/22890)
Supplement: Multimedia Appendix 6 [file mhealth_v9i11e22890_app6.docx]

# **Multimedia Appendix 6: EMA adherence, EMI adherence and Incentives used**

| **Author, year, location** | **EMA adherence** | **EMI adherence** | **Incentive** |
| --- | --- | --- | --- |
| **Mental health** | | | |
| Burns et al. (2011)  USA | NR | NR | NR |
| Bush et al. (2014)  USA | NR | NR | NR |
| Wenze et al. (2016)  USA | Response rate: 58% of all EMAs; user-initiated EMA decreased over time  Time allowed to answer prompt: 12 mins (able to delay for 30 min)  Average time to respond: 14.25 (SD = 20.07) minutes  Time spent per prompt: 2.90 (SD = 1.86) minutes | NR | Received $1 for each of the 120 scheduled momentary assessments they completed |
| Shrier et al. (2017)  USA | NR | NR | Participants received a $10 gift card to a choice of vendors and payment for transportation |
| Bakker et al. (2018)  Australia | NR | NR | NR |
| Kreyenbuhl et al. (2019)  USA | Response rate: 85% overall (79% for the EMAs of positive psychotic symptoms, and 75% for the EMAs of medication side effects) | NR | NR |
| Vaessen et al. (2019)  Netherland | NR | NR | NR |
| Hanssen et al.  (2020)  Netherlands | Response rate: 64% of all EMAs | Participants complied to EMI prompts for 49% of the days throughout the study. | All participants were given 150 Euro for study participation. |
| **Smoking cessation** | | |  |
| Businelle et al (2016) and Hebert et al. (2018)  USA | Response rate: 87% of all EMAs  Time allowed to answer prompt: 60 seconds  Time spent per prompt: 2-6 mins | 83% say they implemented the coping strategies; Participants with higher levels of nicotine dependence accessed the Quit Tips feature 2-3 times more frequently than those with lower nicotine dependence. | Those who completed 50%-74% of assessments received a US $40 gift card, those who completed 75%-89% of assessments received a US $80 gift card, and those who completed 90% or more of their assessments received a US $120 gift card |
| Hebert et al. (2020)  USA | Response rate: 84% of all EMAs | NR for EMI but 52% accessed the on-demand medication tips, and 74% participants accessed the on-demand quit tips | Participants received a US $30 gift card for attending and completing each of the first three post-quit visits (i.e., baseline, quit date, and 4 weeks) and US $50 for completing the 12-week post-quit visit. At the 4-week post-quit visit, those who completed 50%-74% of assessments received US $50 in gift cards, 75%-89% of assessments received US $100, and 90% or more of assessments received US $150. |
| **Substance abuse control** | | |  |
| Dulin et al. (2014)  USA | NR | NR for EMI but participant usage decreased substantially overtime, from 28 tool launchings in Week 1 to 5 tool launchings in week 4. | Compensated for completing baseline and 6-week follow-up assessments |
| Leonard et al. (2017)  USA | Response rate: total of 325 reports which included 261 sensor-band triggered and 64 self-initiated reports; made reports on 78 out of a total 292 days (26.71%)  Time spent with app-initiated and user-initiated reports over the study duration: 17.50 min (SD 9.37) | NR | Participants received US $25 for each assessment, $15 for each in-person counselling session, $25 per week for wearing the sensor band 5 or more hours a day (for 5 out of 7 days), and $5 for sending their data electronically every other day |
| Shrier et al. (2018)  USA | Response rate: 63.5% at baseline and 57.1% at 3-month follow-up. Diary response rates were 85.7% at baseline and 71.4% at follow-up. During the intervention phase, response rates were 35.1% of the momentary reports and 57.1% of the diaries. | NR | Remuneration was up to $175 total. Remuneration was graded over the study and commensurate with completion of study assessments, including study visits ($15–25) and EMA reports ($10–15 for responding to at least 50% of prompts and $20–25 for responding to at least 80% of prompts). |
| **Diet and physical activity** | | |  |
| Mundi et al. (2015)  USA | Response rate: 30.7%; decreased over time.  Time allowed to answer prompt: 60 mins  Time spent with EMA over the study duration: 17.4 ± 4.4 mins | NR | None |
| Goldstein (2018), (2020)  USA | Response rate: 62.9%  Time allowed to answer prompt: 90 mins | On average, participants opened 46.91% (SD=26.18) of alerts which corresponded to opening 7.80 (SD=0.77) risk alerts per week. | Participants were compensated with a free WW app subscription and the opportunity to earn up to $180; 50c were deducted for every prompt not answered within 90 minutes |
| Pentikäinen et al. (2019)  Finland | NR | Frequency of EMI use: 2.6 times/day (baseline), 1.8 times/day (end of study)  Decreased over time | The participants were given 4 movie tickets worth 52 euros to compensate their time and effort. |
| Allicock et al. (2020)  USA | Response rate: 75% | NR | Participants received a $30 gift card for each of the three study visits and could earn up to an additional $60 for completing 80% or more of the ecological momentary assessments. |

Abbreviations: NR, not reported
