# Supplementary material for: Smartphone-Delivered Ecological Momentary Interventions Based on Ecological Momentary Assessments to Promote Health Behaviors: Systematic Review and Adapted Checklist for Reporting Ecological Momentary Assessment and Intervention Studies
Source: JMIR Mhealth Uhealth. 2021 Nov 19;9(11):e22890. doi: 10.2196/22890 (PMC8663593; doi:10.2196/22890)
Supplement: Multimedia Appendix 7 [file mhealth_v9i11e22890_app7.docx]

# **Multimedia Appendix 7: Behaviour Change Techniques present in the interventions of included studies**

| **Behaviour change techniques** | **Frequency** | **Studies** |
| --- | --- | --- |
| 3.1. social support (unspecified) | 13 | Dulin, Leonard, Vaessen, Burns, Goldstein, Wenze, Businelle, Hebert, Shrier (2017), Mundi, Kreyenbuhl, Shrier (2018), Allicock |
| 7.1. prompt cue | 10 | Burns, Bush, Kreyenbuhl, Shrier (2017), Hassen, Mundi, Goldstein, Allicock, Dulin, Vaessen |
| 1.2. problem solving | 9 | Dulin, Leonard, Burns, Bakker, Goldstein, Wenze, Businelle, Hebert, Hassen |
| 2.2. feedback of behaviour | 6 | Leonard, Wenze, Mundi, Kreyenbuhl, Hassen, Pentikäinen |
| 2.3. self-monitoring (behaviour) | 6 | Dulin, Burns, Wenze, Shrier (2018), Kreyenbuhl, Pentikäinen, Allicock |
| 2.4. self-monitoring (outcome) | 6 | Dulin, Bakker, Shrier (2018), Kreyenbuhl, Hassen, Bush |
| 3.3. social support (emotional) | 6 | Dulin, Leonard, Bakker, Wenze, Shrier (2018), Shrier (2016) |
| 4.1. instruction on how to perform behaviours | 5 | Dulin, Vaessen, Burns, Wenze, Mundi |
| 1.4. action planning | 4 | Leonard, Vaessen, Burns, Goldstein |
| 8.1. behavioural practice/ rehearsal | 4 | Vaessen, Burns, Bakker, Wenze |
| 8.2. behaviour substitution | 4 | Dulin, Burns, Mundi, Shrier (2016) |
| 5.1. information about health consequences | 4 | Wenze, Businelle, Hebert, Mundi |
| 1.1. goal setting (behaviour) | 3 | Leonard, Vaessen, Bakker |
| 11.2. reduce negative emotions | 3 | Goldstein, Businelle, Hebert |
| 2.7. feedback of outcome | 2 | Dulin, Shrier (2018) |
| 4.2. information about antecedent | 2 | Leonard, Shrier (2018) |
| 12.3. avoidance | 2 | Dulin, Goldstein |
| 5.3. information about social & environmental consequences | 2 | Businelle, Hebert |
| 9.1. credible source | 2 | Businelle, Hebert |
| 11.1. phamarcological support | 2 | Businelle, Hebert |
| 1.3. goal-setting (outcome) | 1 | Goldstein |
| 1.5. review of goals | 1 | Vaessen |
| 1.6. discrepancy between current behaviour and goal | 1 | Shrier (2018) |
| 2.6. bio-feedback | 1 | Leonard |
| 5.4. information about emotional consequences | 1 | Bakker |
| 5.6. information about emotional consequences | 1 | Bakker |
| 8.4. habit reversal | 1 | Burns |
| 8.6. generalisation of target behaviour | 1 | Vaessen |
| 9.2. pros and cons | 1 | Leonard |
| 10.3. non-specific rewards | 1 | Bakker |
| 12.1. restructuring the physical environment | 1 | Goldstein |
| 12.2. restructuring the social | 1 | Dulin |
| 13.2. framing/ reframing | 1 | Vaessen |
| 13.4. valued self-identity | 1 | Vaessen |
| 15.4. self talk | 1 | Leonard |
