# Supplementary material for: Smartphone-Delivered Ecological Momentary Interventions Based on Ecological Momentary Assessments to Promote Health Behaviors: Systematic Review and Adapted Checklist for Reporting Ecological Momentary Assessment and Intervention Studies
Source: JMIR Mhealth Uhealth. 2021 Nov 19;9(11):e22890. doi: 10.2196/22890 (PMC8663593; doi:10.2196/22890)
Supplement: Multimedia Appendix 8 [file mhealth_v9i11e22890_app8.docx]

# **Multimedia Appendix 8: Behaviour change techniques used in each study and excerpts**

| Author, Year, Country, Health domains | Behaviour change theories used  Behaviour change techniques used |
| --- | --- |
| **Mental health** | |
| Burns et al. (2011)  Mood disorders | **Behaviour change theories used**  Behavioural activation approach  **Behaviour change techniques used**  1.2. Problem solving  1.4. Action planning  2.4. Self-monitoring of outcome of behaviour  3.1. Social support (unspecified)  4.1. Instruction on how to perform behaviour  7.1. prompts/ cue  8.1. Behavioural practice/ rehearsal  8.2. Behaviour substitution  8.4 Habit reversal  **Excerpts:**  1.2., 4.1., 8.2., 8.4. “Examples include an activity calendar with which participants could monitor and schedule their activities, as well as tools leading participants through each step involved in designing behavioural experiments or formulating tailored plans to replace avoidance behaviours with active coping behaviours. These tools were designed to be completed in just a few minutes, and participants were instructed to use the current week’s tool on a daily basis.”  1.4. “Activity calendar with which participants could monitor and schedule their activities”  1.2., 4.1. “Tools leading participants through each step involved in designing behavioural experiments or formulating tailored plans to replace avoidance behaviours with active coping behaviours”  2.4. “both participants and coaches could visualize state data entered into the mobile phone application”  3.1., 7.1. “when a user’s self-reported mood was outside her or his typical range, a message would appear on the phone designed to reinforce improvement or suggest using a tool in the case of deterioration”  4.1. “taught participants how to monitor the effect of daily behaviours on mood, schedule positive activities, experiment with new responses to distressing situations, recognize and change avoidance patterns (eg, rumination and procrastination), and maintain gains after  treatment.”  8.1. “Each lesson was paired with an interactive tool to provide participants with opportunities to apply the treatment concepts discussed in the lesson” |
| Bush et al.  (2014)  Mood and Anxiety disorders | **Behaviour change theories used**  None mentioned  **Behaviour change techniques used**  2.4. Self-monitoring of outcomes of behaviour  **Excerpts:**  2.4. “T2 Mood Tracker displays user-tailored graphs of any selected combination of specific anchors and categories across time. Users can also create notes documenting daily experience such as medications or therapies, personal events, or stresses that might affect each mood rating” |
| Wenze et al.  (2016)  Bipolar disorder | **Behaviour change theories used**  Cognitive Behavioural Therapy  Health Belief Model  **Behaviour change techniques used**   - 1. problem solving   2.2. feedback on behaviour  2.3. self-monitoring of behaviour  3.1. social support (unspecified)  3.3. social support (emotional)  4.1 Instruction on how to perform a behaviour  5.1. Information about health consequences  8.1. Behavioural practice/ rehearsal  **Excerpts:**  1.2. “potential coping strategies (including personalized strategies identified by participants and programmed into their devices) were presented when participants indicated depressed or elevated mood states”  2.2.“participants were asked about actual adherence behaviours (eg, doses taken, appointments attended) and they again received appropriate feedback”  2.3. “smartphone software presented patients with a series of questions that  assessed for symptoms that are common prodromes in BD (eg, mood, changes in sleep, symptoms, psychomotor agitation or retardation, anxiety, and psychotic)”  3.1. “if participants endorsed suicidal ideation and a plan for how to kill themselves, they were told to contact their provider or go to the nearest emergency room”  3.3. “MyT integrates psychoeducational and cognitive-behavioural principles”  4.1 “Teaching coping strategies to deal with side effects and other discomforts of mood disorders”  5.1 “Psychosocial and psychoeducational interventions in mood disorders have effected positive change on adherence by providing patients with information about their diagnosis and the ways in which treatment works. Patients are also encouraged to discuss perceived costs and benefits of adherence. Non-adherence may be addressed by discussing relevant barriers and using cognitive and behavioural strategies to challenge maladaptive thoughts regarding medication and illness. Patients also discussed perceived susceptibility to illness and consequences of depressive and(hypo)manic relapse.”  8.1 “the research clinician used role-playing exercises to help the  participant practice raising concerns with his or her primary mental health care provider.” |
| Shrier et al.  (2017)  Disruptive, Impulse Control, and Conduct Disorder | **Behaviour change theories used**  - cognitive behavioural therapy  **Behaviour change techniques used**  3.1. social support (unspecified)  3.3. social support (emotional)  7.1. prompts/ cues  8.2. behaviour substitution  **Excerpts:**  3.1., 7.1., 8.2. “When she reports feeling upset on the app survey, she would see a message, praising her for identifying her emotions, reminding her that she might be vulnerable to making unhealthy choices prompting her to consider healthy mood management strategies”  3.3. “ The approach was described as in-person counselling and cognitive-behavioural skills training” |
| Bakker et al. (2018)  Mood disorders | **Behaviour change theories used**  - Cognitive behavioural therapy  - Self-determination theory  - The hook model for user-centred design (Eyal, 2014)  - Acceptance Commitment Therapy  - Dialectical behaviour therapy (DBT; Kliem, Kroger, & Kosfelder, 2010)  - Motivational interviewing  **Behaviour change techniques used**  1.2.  Problem solving  2.4. Self-monitoring of behaviour  3.3. Social support (emotional)  5.4 Monitoring of emotional consequences  5.6. Information about emotional consequences  8.1. Behavioural practice/rehearsal  10.3. Non-specific reward  **Excerpts:**  1.2. “Missions are designed to be real-time coping strategies, achievable in a wide variety of situations”  2.4. “Mission Log documents all past Missions attempted in detail”  3.3. “This paper details the development of a smartphone app, “MoodMission,” that aims to provide evidence-based Cognitive Behaviour Therapy (CBT) strategies for mood- and anxiety-related problems”  5.4 “Rate distress again after Mission”  5.6. “Enhances understanding of cause-and-effect relationship between actions and emotions”  8.1. “Encourage use of the app through personal investment. Internal triggers for repeated engagement”  10.3. “Encourage use of the app via rewards and internal triggers, and positive reinforcement and behavioural conditioning” |
| Vaessen et al.  (2019)  Schizophrenia Spectrum and Other Psychotic Disorders | **Behaviour change theories used**  Acceptance Commitment Therapy  **Behaviour change techniques used**   - 1. Goal-setting behaviour   1.4. Action planning  1.5. Review behaviour goal(s)  4.1. Instructions on how to perform the behaviour  7.1. Prompt/ cues  8.1. Behavioural practice/ rehearsal  8.6. Generalisation of target behaviour  13.2. Framing/ reframing  13.4. valued self-identity  **Excerpts:**  1.1., 1.4. Patients are asked to set a practical, feasible goal for that day, to guide them in their commitment to the chosen value.  1.5. patients are asked to evaluate the extent to which they were able to live in keeping with their chosen value and if they were able to reach their goal for the day.  4.1. “During the sessions, people are introduced to the basic principles of ACT and provided with insights, exercises, and metaphors that help in implementing ACT in their lives”  7.1. “Depending on their response, they receive an exercise that taps into current negative experiences or an exercise to train general ACT principles.”  8.1., 8.6. “patients engage in a mobile intervention that requires the active exercise of ACT principles throughout the day in addition to standard ACT sessions”  13.2. “Cognitive defusion is a core ACT skill that requires substantial exercise. Once patients of the ACT-DL program are able to detach from their thoughts, they can transfer these techniques to thoughts about the self as well and let go of their self-image, which is the topic of the “self as context” module”  13.4. “patients identify three values (e.g., family, career, health) which they feel are not sufficiently reflected in their daily behaviour” |
| Hanssen et al. (2020)  Schizophrenia spectrum disorders | **Behaviour change theories used**  none mentioned  **Behaviour change techniques used**  1.2. Problem-solving  2.2. Feedback on behaviour  2.4. Self-monitoring of outcome(s) of behaviour  7.1. Prompts/cues  **Excerpts:**  1.2., 2.2 “The prompts provided suggestions for a certain activity or behaviour change, depending on the previous ESM answers”  2.4 “Symptoms, social activities and mood were assessed.”  7.1. “The prompts provided suggestions for a certain activity or behaviour change, depending on the previous ESM answers ” |
| **Smoking cessation** | |
| Businelle et al.  (2016)  Smoking cessation  Hebert et al.  (2018), (2020)  Smoking cessation | **Behaviour change theories used**  None mentioned  **Behaviour change techniques used**  1.2. problem solving  3.1. social support (unspecified)  5.1 Information about health consequences  5.3. information about social and environmental consequences  9.1 Credible source  11.1. pharmacological support  11.2 Reduce negative emotions  **Excerpts:**  1.2. “Level 2 messages primarily focused on ways to cope with current lapse triggers (i.e., reported during the current EMA)”  3.1., 5.3. “Message topics were primarily motivational in nature and focused on planning and preparing for the quit attempt and benefits of quitting”  5.1 Third, a “Medications” function/button offered information  (eg, common side effects, quit statistics, use instructions) about smoking cessation medications that were regularly prescribed by the Parkland Hospital Smoking Cessation Clinic.  9.1 “Participants attended one initial orientation and educational session provided by a respiratory therapist”  11.1. “All participants received standard smoking cessation clinic care (ie, group counselling and cessation pharmacotherapy)”  11.2. “The “Quit Tips” function/button accessed a menu of treatment-related messages that focused on general smoking cessation advice, various benefits of quitting, and specific suggestions on how participants might cope with stress, urges, and negative mood” |
| **Substance abuse control** | |
| Dulin et al.  (2014)  Alcohol use | **Behaviour change theories used**  Motivational interviewing  **Behaviour change techniques used**  1.2. problem-solving  2.3. self-monitoring of behaviour  2.4. self-monitoring of outcome(s) of behaviour(s)  2.7. feedback on outcome(s) of behaviour  3.1. social support (unspecified)  3.3. Social support (emotional)  4.1. instructions on how to perform the behaviours  7.1. prompt/ cues  8.2 Behaviour substitution  12.2. restructuring the social environment  12.3. avoidance/ reducing exposure to cues for the behaviour  **Excerpts:**  1.2., 2.3. “immediate coping strategies and monitoring functions for on-going alcohol use and alcohol-related problems”  2.4.” assessment feedback as outline of their progress over time (Weekly reports of progress (e.g., change in drinking, cravings associated with drinking, ways to avoid continued drinking)”  2.7. “assessment feedback as outline of their progress over time (Weekly reports of progress (e.g., change in drinking, cravings associated with drinking, ways to avoid continued drinking”  3.1 “The Supportive Persons Tool allowed users to select, add and delete people who could be trusted to be of help during times of need. ”  3.3. “Combined Behavioural Intervention used in Project Combine: Combined Behavioural Intervention (CBI)- Phase 1 emphasizes building motivation for change. It begins with a single session of motivational interviewing (Miller and Rollnick 1991), which is the general clinical style used throughout CBI. This is followed by client assessment feedback in the style of motivational enhancement therapy (Miller et al. 1992). (extracted from: https://pubs.niaaa.nih.gov/publications/combine/overview.htm)  4.1. “The Problem Management Tool provided step-by-step instruction on how to directly approach a life problem”  7.1. “After high-risk locations were entered, the system utilized the GPS capabilities of the smartphone to provide an audible alert and suggestions for maintaining control of drinking when they crossed a boundary around a specific high-risk location”  8.2 “The Pleasurable Activities Tool provided numerous different categories of non-drinking activities and the ability to schedule them into a calendar.”  12.2., 12.3. “high risk for drinking locations & strategies to avoid them” |
| Leonard et al.  (2017)  Alcohol use | **Behaviour change theories used**  - motivational interviewing approach  - cognitive behavioural therapy  **Behaviour change techniques used**  1.2 problem-solving  1.1 goal-setting (behaviour)  1.4. action planning  2.2. feedback on behaviour  2.6. Biofeedback  3.1. social support (unspecified)  3.3. Social support (emotional)  4.2. information about antecedents  9.2. pros and cons  15.4. Self-talk  **Excerpts:**  1.1 “developing short-terms goals for reducing risky drinking”  1.2 “provide coping strategies based on participant responses”  1.1, 1.4 “If you choose to cut back, what would you want to consider in making your plan (eg, number of drinks and frequency)?”  2.2. “personalized feedback on drinking patterns and motives for drinking”  2.6 “The Empatica E4 wearable wristband (“sensorband”) measures EDA by applying constant low voltage to the skin and measuring the resultant current.”  3.1. “2 in-person alcohol brief intervention counselling sessions”  3.3. “The intervention used motivational interviewing (MI) and cognitive behavioral therapy (CBT) strategies for reducing risks associated with drinking”  4.2. “identifying triggers”  9.2. “Decisional balance”  15.4. “Cool thoughts: my thought is not me” |
| Shrier et al.  (2018)  Drug use | **Behaviour change theories used**  - Screening, Brief Intervention, and Referral to Treatment (SBIRT)  - motivational interviewing  - motivational enhancement therapy  **Behaviour change techniques used**  1.6. Discrepancy between current behaviour and goal  2.3 Self-monitoring of behaviour  2.4. Self-monitoring of outcome(s) of behaviour  2.7. feedback on behaviour  3.1. social support (unspecified)  3.3. Social support (emotional)  4.2. information about antecedents  **Excerpts:**  1.6 “developed discrepancy between use and the values and goals.”  2.3, 2.4 “prompting report of marijuana-related momentary states, contexts, and behavior. Participants also completed self-scheduled daily diaries, summarizing marijuana withdrawal symptoms, cigarette and marijuana use, and motivation to reduce or cease marijuana over the past 24 hours.”  2.7. “participants received and discussed a personalized feedback report on their use”  3.1. “Participants then met with a study counsellor in clinic for two 1-hour MET sessions separated by one week”  3.3. using motivational messages of SBIRT, “often include recommendations for brief motivational interviewing (MI) when problematic substance use is identified”.  4.2. “Participants identified triggers for use from lists of affective states and social contexts” |
| Kreyenbuhl et al.  (2019)  Medication adherence | **Behaviour change theories used**  Information-Motivation- Behavioural (IMB) Skills Model  **Behaviour change techniques used**  2.2. feedback on behaviour  2.3. self-monitoring of behaviour  2.4. self-monitoring of outcome(s) of behaviour  3.1. Social support (unspecified)  7.1. prompts/ cues  **Excerpts:**  3.1, 2.2. “tailored motivational feedback to encourage adherence”  2.3., 2.4. “stores and tracks information about their self-reported medication adherence, positive psychotic symptoms, and antipsychotic side effects”  7.1. “automated reminder for medication administration” |
| **Diet and physical activity** | |
| Mundi et al.  (2015)  Education and engage in healthy lifestyles to prepare for bariatric surgery | **Behaviour change theories used**  None mentioned  **Behaviour change techniques used**  2.2. feedback on behaviour  3.1. social support (unspecified)  4.1. instructions on how to perform a behaviour  5.1. information about health consequences  7.1. prompts/ cues  8.2. behaviour substitution  **Excerpts:**  2.2. “Frequency of eating/snacking Frequency of use of calorie-containing beverages.”  3.1., 8.2 “if a patient endorsed a healthy lifestyle behaviour (i.e., reduced consumption of calorie-containing beverages), they were sent a congratulatory/supportive message. Alternatively, if a patient was struggling to make a positive lifestyle modification, they were sent a supportive message outlining some alternative strategies”  4.1., 5.1. “The smartphone app also housed nine short (3 to 9 min in length) video-based education modules, which were followed by a linked assessment to verify mastery of topic”. The topics include: Diet hygiene, Food labels, Restaurant menus, Servings and portions, Your diet after surgery, Physical activity-related modules, Exercise basics, Getting started, Your personal fitness plan, Maintaining motivation  7.1. “Reduction of distraction while eating Timing/slowing rate of eating”. |
| Goldstein  (2018)  Diet lapse | **Behaviour change theories used**  Principles for behaviour change (Abraham & Michie, 2008)  **Behaviour change techniques used**  1.2. Problem solving  1.3. goal setting (outcome)  1.4. action planning  7.1. prompts/ cues  11.2. Reduce negative emotions  12.1. Restructuring the physical environment  12.3. Avoidance/reducing exposure to cues for the behaviour  **Excerpts:**  1.2.,7.1. an alert was issued communicating (a) that the participant was at risk, the top three factor(s) substantially contributing to level of risk (e.g., extracted from the optimized machine learning algorithm), and (c) possible strategies to cope with each specific risk factor derived from  1.3., 1.4. “Commercial Weight Watchers (WW): mobile program serves as the dietary prescription for weight loss (i.e., daily point goals that correspond to caloric intake)”  11.2 “Interventions typically addressed different lapse triggers via cognitive (e.g. restructuring negative thoughts)”  12.1, 12.3 (e.g. avoid problematic locations) strategies. |
| Pentikäinen et al.  (2019)  Diet lapse | **Behaviour change theories used**  Self-determination  **Behaviour change techniques used**  2.2 Feedback on behaviour  2.3. self-monitoring of behaviour  **Excerpts:**  2.3. The Button comprises 2 components: the desktop widget and the actual app. The user presses 1 of the 2 buttons reflecting different types of eating occasions (healthy or unhealthy or content or discontent) in the Button widget after each eating occasion to record the time stamp and type of the eating occasion.  2.2 “The Button app visualizes the user’s eating pattern with 3 summary screens.” |
| Allicock et al. (2020)  Physical activity and diet adherence | **Behaviour change theories used**  Social cognitive theory and control theory  **Behaviour change techniques used**  2.3. self-monitoring of behaviour  3.1. social support (unspecified)  7.1. Prompts/cues  **Excerpts:**  2.3., 3.1., 7.1.Strategies from the social cognitive theory and control theory recommend providing individuals opportunities to self-monitor and regulate their behaviour, providing behavioural cues/prompting, increasing self-efficacy, building behavioural capability, and providing positive reinforcements to behaviours further predicts behavioural achievement and adherence. The messages incorporated these theoretical aspects to encourage behaviour engagement. |
