# Supplementary material for: Smartphone-Delivered Ecological Momentary Interventions Based on Ecological Momentary Assessments to Promote Health Behaviors: Systematic Review and Adapted Checklist for Reporting Ecological Momentary Assessment and Intervention Studies
Source: JMIR Mhealth Uhealth. 2021 Nov 19;9(11):e22890. doi: 10.2196/22890 (PMC8663593; doi:10.2196/22890)
Supplement: Multimedia Appendix 9 [file mhealth_v9i11e22890_app9.docx]

# **Multimedia Appendix 9: User perspectives and suggestions**

| **Author (Year)**  **Health domains** | **User perspectives and suggestions** |
| --- | --- |
| Burns et al. (2011)  Mood disorders management | **Negative perspectives**  - Numerous technical problems related to battery drainage and connectivity  - Issues with interpretation of sensor data  **Suggestions**  - Additional features needed are:   - More interactive features, like a blog and a tool to allow voice recording of affective states. - Communication/messaging with coaches - Personalised tools to allow users to create individually tailored states. |
| Bush et al. (2014)  Mood and anxiety disorders management | **Positive perspectives**  - Most users said the app was easy to use and they would like to continue using the app in the future  - Multimedia capability of the app was especially useful  **Negative perspectives**  Negative comments were few, mostly on technical issues.  **Suggestions**  - Ability to communicate with a clinician provider if user’s mood is declining  - Visual appeal/design are important |
| Wenze et al. (2016)  Bipolar disorder management | **Positive perspectives**  - High credibility score (22.4/27) and moderate expectancy to change (19.75/27) on Credibility and Expectancy Scale  - High level of satisfaction (4.63/5), perceived helpfulness (4.88/5), ease of use (4.63/5)  - All participants reported that EMI sessions were useful in at least 1 important way (eg, as a source of extra support, increasing insight/awareness of behaviour patterns, improving adherence)  **Negative perspectives**  - Technical issues  - Repetitive content  **Suggestions**  - Future interventions should:   - Greater personalisation, allowing participants to schedule their own EMI sessions - Greater variation in smartphone-delivered feedback and responses |
| Shrier et al. (2017)  Impulse control disorder management | **Positive perspectives**  - Random prompts increased affect recognition and helped when emotions might have interfered with initiating use of the app  - The messages were timely, comforting, and supportive, while brief and immediate  - Reporting/data entry was simple and intuitive (ease of use)  **Suggestions**  - Personalisation is important  - Different options to record data, including multiple choice, free text, voice recording  - Improve visual appeal  - Provide multiple suggestions to improve mood (less repetitive content) |
| Bakker et al. (2018)  Mood and anxiety disorders management | **Positive perspectives**  - Users enjoyed the missions, particularly their speed, ease of use, and emotional relevance  - Mood Missions was rated highly compared to other apps in areas of entertainment, aesthetics, and information  - Non-clinical language was helpful in making the app more accessible  **Negative perspectives**  - EMA prompts/surveys too long and tedious to complete  - Some features not easy to be understood |
| Kreyenbuhl et al. (2016)  Promote antipsychotic medication adherence | **Positive perspectives**  - Users reported that the app was easy to use, pleasant yet challenging, but not stressful, and did not interfere negatively with daily activities  **Negative perspectives**  - (57%) “strongly agreed” or “agreed” that they needed support for technical issues |
| Vaessen et al. (2019)  Psychotic disorders management | **Negative perspectives**  - The total number of EMA prompts per day and the amount of time required to fill out each questionnaire were moderately burdensome  - EMA might lead to an unpleasant degree of self-awareness  **Suggestions**  - Reduce EMA burden  - Tailor intervention based on user’s responses (personalisation) |
| Hanssen et al. (2020)  Schizophrenia spectrum disorders management | **Positive perspectives**  - 94% users reported that the app was easy to use and the design was appealing  - 68% users reported that the app helped them to reflect on their experiences and increase awareness of their behaviour through the prompted questions  - 54% users found coping tips useful  - Loneliness reduced as participants felt the app as someone listening to them  **Negative perspectives**  - Users sometimes received feedback that was not relevant at the time that they received it  **Suggestions**  - Integrating mobile sensing, useful to detect changes in activity levels in an objective way  - Adding personalised video feedback |
| Businelle et al. (2016) and Hebert et al. (2018)  Smoking cessation and relapse prevention | **Positive perspectives**  - 97% users rated app-generated messages as helpful  - 90% reported that the app made them more aware of their thoughts, feelings, and behaviours  - 75% rated EMA frequency prompted by the app was “about right” (75%)  - 83% participants used the app automatic tailored/personalised messages during the post quit period  - 97% users would use the app in the future and 85% would recommend it to friends |
| Hebert et al. (2020)  Smoking cessation and relapse prevention | **Positive perspectives**  - 70.8% of participants in all 3 arms agreed that they can rely on the treatment to provide guidance to help them quit and maintain cessation and 69.2% believed that the treatment would help them quit and maintain cessation.  - Most participants thought the app was not burdensome.  - Participants in intervention groups interacted most frequently with features that offered general quitting advice, as well as features that helped manage their stress and mood.  **Suggestions**  - Personalisation may increase acceptability of smartphone-based cessation interventions |
| Dulin et al. (2014)  Alcohol abuse treatment | **Positive perspectives**  - 84% users said the app helped them enhance awareness about their alcohol consumption  - Tools related to managing alcohol cravings, monitoring consumption, identifying triggers to drink, and managing difficulties in treatment for alcohol use disorder were rated by participants as particularly helpful  - The app was easy to comprehend and use  **Negative perspectives**  - 61% said they would have used the system more if the app were on their phone or another type of phone  - 49% reported on the bad functioning of the location tool (technical issues)  **Suggestions**  - Important areas to improve: personalisation, reduce invasiveness and enhance privacy  - Maximize the quality of user interface and design |
| Leonard et al. (2017)  Alcohol abuse prevention and management | **Positive perspectives**  - High levels of acceptability (3.4/4 of Client Satisfaction Questionnaire)  - Technology component “somewhat” or “very easy” to learn and use, although there was significant variability in the level of satisfaction with the sensor-band and the app.  **Negative perspectives**  - Technical issues (connectivity problems)  - Inopportune alerts/prompts  - 30% users said that alerts sometimes increased their anxiety or level of stress |
| Shrier et al. (2018)  Marijuana use cessation | **Positive perspectives**  - More than 93% users had overall high opinion of the quality of support received  - Highly rated acceptability; comments reflected changing motivation and behaviour  - Easy to use, clear and understandable  **Negative perspectives**  - The smartphone prompts were too frequent (54%), annoying (49%), or burdensome (32%).  - Repetitive messages |
| Mundi et al. (2015)  Promote healthy lifestyles to prepare for bariatric surgery | **Positive perspectives**  - High satisfaction with the app  - 60% users reported that app fit into their routine somewhat easily or very easily  - 40% users said the amount of EMA messages was perfect  **Suggestions**  - Many subjects reported an inability to answer a given EMA message in the 60-min window => snooze feature was requested  - Reminders or push notifications regarding module completion |
| Goldstein (2018) (2020)  Diet adherence | **Negative perspectives**  - Prompts to complete surveys were monotonous and perceived as less helpful, repetitive and more tedious to complete over time  - Repetitive EMI  - Highly frequent alerts and inaccurate risk alerts contributed to feelings that the app was not tailored enough  **Suggestions**   - More personalisation of prompts - Release new content semi-regularly instead of having all available at once to maintain engagement |
| Pentikäinen et al. (2019)  Diet adherence | **Positive perspectives**  The app helped users gain insights into the eating rhythm or eating habits, thus increased awareness on their eating patterns. |
| Allicock et al. (2020)  Promote physical activity and diet adherence | **Positive perspectives**  - 76% users reported that health information via smartphone was extremely or very helpful  - 84% users reported that information was completely or very much so applied to their life.  - 81% users reported the number of smartphone assessments was just right  **Suggestions**  - Higher interactivity and more components (e.g. communication with a coach/dietitian for dietary support, cooking demonstrations, suggesting affordable at-home physical activity options, such as walking groups and exercise classes close to participants’ homes) |
